# Zeb2 DNA-Binding Sites in Neuroprogenitor Cells Reveal Autoregulation and Affirm Neurodevelopmental Defects, Including in Mowat-Wilson Syndrome

**DOI:** 10.3390/genes14030629

**Published:** 2023-03-02

**Authors:** Judith C. Birkhoff, Anne L. Korporaal, Rutger W. W. Brouwer, Karol Nowosad, Claudia Milazzo, Lidia Mouratidou, Mirjam C. G. N. van den Hout, Wilfred F. J. van IJcken, Danny Huylebroeck, Andrea Conidi

**Affiliations:** 1 Department of Cell Biology, Erasmus University Medical Center, 3015 Rotterdam, The Netherlands; 2 Center for Biomics-Genomics, Erasmus University Medical Center, 3015 Rotterdam, The Netherlands; 3 Department of Biochemistry and Molecular Biology, Medical University of Lublin, 20-093 Lublin, Poland; 4 The Postgraduate School of Molecular Medicine, Medical University of Warsaw, 02-091 Warsaw, Poland; 5 Department of Development and Regeneration, KU Leuven, 3000 Leuven, Belgium

**Keywords:** chromatin immunoprecipitation sequencing, embryonic stem cells, Mowat-Wilson syndrome, neural differentiation, neurodevelopmental disorder, syndromes, target genes, transcription factor, transcriptomics, Zeb2

## Abstract

Functional perturbation and action mechanism studies have shown that the transcription factor Zeb2 controls cell fate decisions, differentiation, and/or maturation in multiple cell lineages in embryos and after birth. In cultured embryonic stem cells (ESCs), Zeb2’s mRNA/protein upregulation is necessary for the exit from primed pluripotency and for entering general and neural differentiation. We edited mouse ESCs to produce Flag-V5 epitope-tagged Zeb2 protein from one endogenous allele. Using chromatin immunoprecipitation coupled with sequencing (ChIP-seq), we mapped 2432 DNA-binding sites for this tagged Zeb2 in ESC-derived neuroprogenitor cells (NPCs). A new, major binding site maps promoter-proximal to *Zeb2* itself. The homozygous deletion of this site demonstrates that autoregulation of *Zeb2* is necessary to elicit the appropriate Zeb2-dependent effects in ESC-to-NPC differentiation. We have also cross-referenced all the mapped Zeb2 binding sites with previously obtained transcriptome data from Zeb2 perturbations in ESC-derived NPCs, GABAergic interneurons from the ventral forebrain of mouse embryos, and stem/progenitor cells from the post-natal ventricular-subventricular zone (V-SVZ) in mouse forebrain, respectively. Despite the different characteristics of each of these neurogenic systems, we found interesting target gene overlaps. In addition, our study also contributes to explaining developmental disorders, including Mowat-Wilson syndrome caused by *ZEB2* deficiency, and also other monogenic syndromes.

## 1. Introduction

Zeb2 (also named Sip1/Zfhx1b) and Zeb1 (δEF1/Zfhx1a), the two members of the small family of Zeb transcription factors (TFs) in vertebrates, bind to DNA to two separated E-box like sequences, as determined by in vitro binding to double-stranded oligonucleotides and/or Zeb-mediated repression of transfected reporter DNA constructs [1,2,3,4]. Spaced bi-partite CACCT sequences, often present as CACCTG E2-box, and sometimes CACANNT(G) sequences [1,2], are bound via two (between Zeb1 and Zeb2) highly conserved, separated clusters of zinc fingers [3]. Mutations in *ZEB2* cause Mowat-Wilson Syndrome (MOWS, OMIM#235730) [5,6,7], a rare congenital disease. MOWS patients display intellectual disability, epilepsy/seizures, typical facial dimorphism, and often Hirschsprung disease (HSCR), as well as multiple other defects [8,9,10]. Typical are also the delay in developmental milestones such as motoric development, and anomalies of eyes and teeth. Other features include specific craniofacial malformation and sensorineural deafness, which together with HSCR originate from defects in the ZEB2-positive (+) cells of the embryonic neural crest cell lineage. Meanwhile, mutant *ZEB2* alleles have been determined for about 350 patients [11,12,13,14,15,16]. Recent reports have described malformations in the central nervous system of MOWS patients over a broad age range, including defects of the corpus callosum and/or hippocampus, and can be seen by neuroimaging. These reports also followed up on electro-clinical defects, such as focal seizures, with MOWS patients [16,17,18].

Zeb2’s multiple functions, its action mechanisms and many partner proteins, the still few proven or candidate direct target genes, and lists of genes whose normal expression depends on intact Zeb2 levels, have been documented in various cell types [19,20,21,22,23,24,25,26,27,28,29]. For a recent review, see [30]). Such combinations of studies have allowed for explanations of specific phenotypes caused by Zeb2 perturbation. Zeb2 DNA-binding to candidate target genes helped to explain *Zeb2* loss-of-function phenotypes in embryonic stem cells (ESCs) and initial cells of early and late embryos, later followed by post-natal and adult mice. These intact-Zeb2 dependent and/or Zeb2 target genes are involved in pluripotency (*Nanog*, *Sox2*), cell differentiation (*Id1*, *Smad7*), and embryonic brain cortical and adult neurogenesis (*Ntf3*, *Sox6*), as well as in epithelial-to-mesenchymal transition (EMT) (*Cdh1*) [19,20,21,22,23,24,25,26]. In reverse, subtle mutagenesis of Zeb2 DNA-binding sites in demonstrated target genes also confirmed Zeb DNA-binding, including its repressive activity on mesodermal X*Bra* in *Xenopus* embryos [27] and *Cdh1* in epithelial cells [19].

Despite its critical functions in the precise spatial-temporal regulation of expression of many system/process-specific relevant genes during embryogenesis and post-natal development, and more recently adult tissue homeostasis, stem cell-based repair, and acute and chronic disease [28,29,30], data from chromatin immunoprecipitation followed by sequencing (ChIP-seq) for Zeb2 has been obtained in very few cases only. A major reason is that ChIP-seq grade antibodies specific for Zeb2 are not readily available. ChIP-seq data have been published for high-Zeb2 hepatocellular carcinoma and leukemia cell lines, or cultured cells that overproduce an epitope-tagged Zeb2 (tag-Zeb2) from cDNA-containing episomal vectors or the safe-harbor *Rosa26* locus [25,31,32]. Neither of these represent normal endogenous Zeb2 levels and dynamics. Furthermore, many anti-Zeb2 antibodies cross-react with Zeb1, so do not discriminate between both proteins when their presence overlaps or succeeds one another in a given cell type. However, these TFs compete for the same target genes, which for the individual proteins in any case, depends on cell identity/state, extrinsic stimulation of the cells, or cellular context (e.g., as demonstrated in somitogenesis [33] and melanoma [34]).

In undifferentiated mouse (m) ESCs, Zeb2 mRNA/protein is undetectable, whereas during neural differentiation (ND) of ESCs its strong upregulation accompanies efficient conversion of naïve ESCs into epiblast stem cell like cells (EpiLSCs). This is essential for the subsequent exit of ESCs from primed pluripotency and the onset of ESC differentiation, including progression to neuroprogenitor cells (NPCs) [25]. Here, we have edited one *Zeb2* allele of mESCs by inserting a Flag-V5 epitope tag just before the Zeb2 stop codon, in-frame with the last exon (ex9 of mouse *Zeb2* [35]). These Zeb2-V5 mESCs were then differentiated into NPCs and the Zeb2 DNA-binding sites were determined by V5-tag-based ChIP-seq. Doing so, we identified 2432 binding sites for Zeb2 in NPCs, of which 2294 map to 1952 protein-encoding genes. We then cross-referenced these ChIP-positive (+) target genes with RNA-sequencing data of differentially expressed genes (DEGs) in cell-type specific, neurodevelopment-relevant Zeb2 perturbations [23,36,37]. Although we compare non-identical systems, the overall approach still revealed a number of interesting overlaps of target genes, as well as Zeb2’s role in regulating critical targets in neurodevelopment, including its own gene promoter. Taken together, for the first time we report the identification of Zeb2’s genome-wide binding sites (GWBS) in ESC-derived NPCs at normal Zeb2 level.

## 2. Materials and Methods

### 2.1. ESC Culture Conditions and Differentiation

CGR8 (strain 129) wild-type (WT) and Zeb2-Flag-V5+ (in brief, Zeb2-V5) mouse (m) ESCs were cultured and differentiated towards the neural lineage [38], with few modifications). Briefly, the mESCs were cultured on 0.1% gelatin-coated plates in ESC-medium (DMEM supplemented with 15% heat-inactivated (HI) foetal bovine serum (FBS), 2 mM L-Glutamine, 1× Non-Essential Amino Acids (NEAA), 143 µM β-mercapto-ethanol (β-EtSH) (all ThermoFisher Scientific, TFS, Waltham, MA, USA) and leukemia inhibitory factor (LIF) at 10^3^ U/mL).

For neural differentiation (ND), 4 × 10^6^ cells were plated on non-adherent 10-cm dishes (Greiner, Kremsmünster, Austria) and allowed to form cellular aggregates (CAs) in 10 mL CA-medium (DMEM, 10% HI-FBS, 2 mM L-Glutamine, 1× NEAA, and 143 µM β-EtSH). From day (D) 4 of ND, cells were grown in CA-medium supplemented with 5 µM retinoic acid (RA). During the aggregation stages of ND, the medium was changed every other day by carefully collecting the aggregates with a 10-mL pipet and transferring them to a 15-mL conical tube. The CAs were allowed to sink to the bottom of the tube where, after the previous medium was carefully discarded, the CAs were then gently resuspended in fresh medium and transferred back to the dishes. At D8 of ND, the aggregates were harvested and dissociated by resuspension in 1 mL Accutase (TFS) and pipetting them up and down using a 1-mL pipet, after shaking them in a 37 °C water bath for 5 min. The Accutase was deactivated by adding 9 mL of fresh N2-medium (DMEM with 2 mM L-Glutamine, 50 µg BSA/mL, and 1× N2-supplement) to the dissociated cells, and pelleting the cells gently for 5 min at 200 g. The cells were then resuspended in fresh N2-medium. To ensure single-cell suspension, the cells were filtered by passing them through a 40-μm nylon cell strainer (Corning, Corning, NY, USA); 2.5 × 10^5^ cells/cm^2^ were then plated on poly-DL-ornithine hydrobromide/laminin (both from Sigma-Aldrich, St. Louis, MI, USA) coated plates. Cells were harvested at D8 or D10 of ND.

### 2.2. Western Blots

To check for Zeb2-V5 protein, the 2BE3-clone ESCs were subjected to ND till D8. Cytoplasmic and nuclear fractions were split using the NePer-kit^®^ (TFS). Protein concentrations were measured using the Bradford BCA (TFS), and equal quantities of protein lysates were loaded on 6% polyacrylamide gels (with SDS) and thereafter cut, according to relative molecular mass. Gels were then transferred onto nitrocellulose membranes (Amersham Bioscience, Amersham, UK), which were incubated overnight with anti-Zeb2 [20] and anti-V5 (Life Technologies, Carlsbad, CA, USA) antibodies, followed by incubation at room temperature with horse radish peroxidase (HRP) conjugated secondary anti-rabbit and anti-mouse antibodies (Jackson ImmunoResearch, West Grove, PA, USA). Protein bands corresponding to Zeb2 or Zeb2-V5 were visualized on an AI-600 digital imager (Amersham Bioscience). As loading control, we used Valosin-containing Protein (VCP) and anti-VCP antibody (Santa Cruz, Dallas, TX, USA, sc-57492, mouse).

### 2.3. RNA Extraction and RT-qPCR Analysis

Total RNA was extracted from ESCs using TRI Reagent (Sigma), and used for cDNA synthesis with RevertAid RT Kit (from TFS) with oligodT-primers. RT-qPCR was performed using SybrGreen dye (BioRad, Hercules, CA, USA) on a CFX96 T1000 thermal cycler (BioRad). All data shown are averages of three independent biological replicates and three technical replicates, normalized to β-Actin mRNA levels. Primers are listed in Table 1. Analysis and data visualization was performed in R environment for statistical computing version 3.5.3, implemented with the tidyverse v1.3 package (https://github.com/tidyverse, accessed on 1 November 2019).

### 2.4. Tag-Zeb2 Mouse ESCs

gRNAs (Table 2) targeting *Zeb2*-ex9, and tracrRNA (Integrated DNA Technologies, IDT, Coralville, IA, USA), were diluted to 125 ng/µL in duplex buffer (from IDT). gRNAs were annealed to tracrRNA at a 1:1 ratio at 95 °C for 5 min and cooling the samples to room temperature. 250 ng of these annealed gRNAs were transfected in 350,000 mESCs together with 2 µg pX459-Cas9-puro vector and 1 µg ssDNA oligo of the donor template containing the FlagV5-tag sequence (Table 2). Transfection was done in a gelatin-coated 6-well plate using DNA: Lipofectamine-2000 (ratio of 1:2). Six hours after transfection the medium was refreshed, and at 24 h the cells were selected in puromycin (2 µg/mL). After two days, the remaining cells were transferred to gelatin-coated 10-cm dishes and given fresh ESC medium (see below). Per dish 1000; 1500; or 2000 cells were plated and allowed to form colonies. The medium was changed every other day. Colonies were picked, expanded, and genotyped by PCR (both outer and inner primer sets were used (Table 1; Figure S1). All candidate clones were validated by Sanger-sequencing; correct clones were expanded and validated by western blot. Mouse ESCs genome-editing was performed under the GGO (genetically modified organisms) institutional licenses 95-053 and 99-164 assigned to the Erasmus University Medical Center.

### 2.5. CRISPR/Cas9-Mediated Deletion of the Zeb2 Binding Site Located at chr2:45109746-45110421

Oligonucleotides for gRNAs (Table 2) with target outside of this chr2-region were cloned into *Bbs*I-digested pX330-hspCas9-T2A-eGFP plasmid. All resulting plasmids used further were sequenced. 4 µg of gRNA-plasmids (1 µg each) were transfected in 350,000 mESCs and selected (see above). After 24 h these cells were sorted as GFP+ cells (LSR Fortessa, Becton-Dickinson (BD), Franklin Lakes, NJ, USA). Per well of a 6-well plate 1000; 1500; or 2000 GFP+ cells were plated and colonies were allowed to form, picked (see above), and genotyped by PCR using primers flanking this deletion, and within and outside of it. Clones showing a possible heterozygous or homozygous deletion, as concluded from the PCR analysis, were subjected to ND. At D8, they were harvested, RNA was isolated and cDNA synthesized (see below), and amplified (for the primers, see Table 1). All candidate clones were validated by Sanger-sequencing.

### 2.6. Chromatin Immunoprecipitation (ChIP)

ChIP 2 × 10^8^ cells were harvested at ND-D8 in 10 mL of PBS and cross-linked using 1% formaldehyde (Sigma Aldrich) for 15 min, rotating at room temperature. Quenching followed with 125 mM glycine for 5 min, again rotating at room temperature. Cross-linked cells were washed twice with ice-cold PBS (5 min; 1500 rpm (240 rcf), 4 °C) before the pelleted cells were snap-frozen and stored at −80 °C. For sonication, the cell pellets were thawed on ice, resuspended in 1 mL sonication buffer (10 mM Tris-HCl pH 8.0, 1 mM EDTA, 0.5 mM EGTA) supplemented with protease and phosphatase inhibitors (PPI, from Roche, Basel, Switzerland), and incubated on ice for 10 min. DNA was sheared by sonicating the cells using a probe sonicator (32 cycles, 30 sec-on amplitude 9, and 30 sec-off). These samples were centrifuged at 13,200 rpm (17,000 rcf) for 10 min at 4 °C. Chromatin pellets were snap-frozen and stored at −80 °C. To check sonication efficiency, 50 µL of sample was de-crosslinked overnight by adding NaCl (final concentration 5 mM) at 65 °C and constantly shaken (950 rpm, ThermoMixer C, Eppendorf, Hamburg, Germany). The next morning 5, 10, and 20 µL of sample were loaded on a 2% agarose gel, revealing ideally a DNA-smear around 300 bp. A 50-µL sample was used as a control input.

For immunoprecipitation, the chromatin of 10^7^ cells was diluted in ChIP-dilution buffer (17 mM Tris-HCl pH 8.0, 170 mM NaCl, 1.2 mM EDTA, 0.01% SDS, 1.1% Triton X-100, with 1× PPI) to a final volume of 1 mL. Samples were pre-cleared by adding pre-washed Protein A/G agarose beads (Santa Cruz) and further incubation for 1 h, rotating at 4 °C. Then, samples were centrifuged for 1 min (1000 rpm; 106 rcf) at 4 °C, and the pre-cleared chromatin (supernatant) was transferred to a new low-binding 1.5-mL tube and incubated with 50 µL of pelleted V5-Agarose beads (Sigma Aldrich), rotating at 4 °C overnight. Before the addition of V5-agarose beads, the beads were washed 5 times (5 min each) in PBS by rotating them. As a negative control, half of the sample was incubated with Protein A/G beads (Santa Cruz, sc-2003).

The following day the beads were pelleted (1000 rpm; 1 min) and washed as follows: once with lower-salt buffer (20 mM Tris-HCl pH 8.0, 150 mM NaCl, 2 mM EDTA, 0.1% SDS, 1% Triton X-100), transferred to non-stick low-binding 1.5-mL tubes and then washed once with high-salt buffer (i.e., lower-salt buffer, but now 500 mM NaCl), once washed with LiCl buffer (which is 10 mM Tris-HCl pH 8.0, 250 mM LiCl, 1 mM EDTA, 1% NP-40, 1% sodium deoxycholate (DOC), and twice washed with 10 mM Tris-HCl pH 8.0, 1 mM EDTA (each incubation for 5 min, rotating at 4 °C, followed by gently spinning down.

The protein-chromatin was then eluted from the beads by adding 250 µL of elution buffer (1% SDS, 100 mM NaHCO_3_), rotating for 1 h at room temperature twice, and combining the eluates from both steps. To the input sample, 450 µL of elution buffer was also added, and all samples were de-crosslinked through the addition of 5 mM NaCl at 65 °C overnight, shaking at 950 rpm. The day after, 2 µL of proteinase-K (from 10 mg/mL stock), 20 mM (final concentration) Tris-HCl pH 6.5, 5 mM (final concentration) EDTA pH 8.0 and 10 mg/mL RNase-A (Sigma-Aldrich) were added to each sample and incubated for 1 h at 45°C while shaking (700 rpm). DNA was extracted from the samples using the PCI method and diluted in water. Five independent ChIPs were performed, for a total of 10^8^ mESCs used per condition, and pulled-down chromatin was pooled. ChIP efficiency was assessed by qPCR using primers amplifying *Cdh1* promoter sequences bound by Zeb2 [25]. All primers used are listed in Table 1.

### 2.7. ChIP-Sequencing

DNA libraries from input (i.e., control) and V5 ChIPs were prepared using ThruPLEX DNA protocol (TakaraBio, Kusatsu, Shiga, Japan) specific for low amounts of DNA and sequenced on Illumina HiSeq-2500, and single reads of 50 bp were generated. Adapter sequences were trimmed from the 3′-end of the reads, after which the reads were aligned to the mm10/GRCm38 genome using HISAT2 [39]. From the alignments, secondary or supplementary, low-quality, and fragmented alignments (fragments longer than 150 bp) were filtered away. Peaks were called with MACS [40], and coverage was determined. 42 and 25 million reads were generated for input and V5 ChIP, respectively.

### 2.8. ChIP-Sequencing Data Analysis

Peak calling was performed with MACS2 (Galaxy version 2.1.1.20160309.6) [40,41], with default parameters (narrow peak calling, Mm1.87e9, FDR < 0.05) using the input sample as background. The No model parameter was used, and the extension size was set at 210 bp based on the predicted fragment lengths from the alignments (MACS2 predict-tool, Galaxy version 2.1.1.20160309.1) [40,41]. The distance of the aligned reads from the TSS of the gene was analyzed using ComputeMatrix (Galaxy version 3.3.2.0.0) and PlotHeatmap Galaxy version 3.3.2.0.1; the used matrix is based on the log2ratio of the aligned ChIP peaks over the input, calculated using BamCompare (Galaxy version 3.3.2.0.0) [42].

### 2.9. Transcription Factors Motif Enrichment Analysis

To identify the transcription factor binding sites (TFBS) in Zeb2-binding regions associated with DEGs, we first extracted unique Zeb2-peaks located 10 kb −/+ from the transcription start site (TSS). Next, we analyzed the TFBS enrichment using a UniBind enrichment tool with motifs from the UniBind database [43] (using reference genome GRCm38/mm10). As a background for the analysis, all Zeb2-peaks were used. The *p*-value from Fisher’s exact test after multitest adjustments was used to identify significantly enriched TFBS. Further, the max rank index calculated based on the odds ratio, *p*-value from Fisher’s exact test, and the number of overlapping regions, was applied to rank the top enriched motifs.

### 2.10. RNA-Sequencing

The quality of total RNA (of biologically independent triplicates) of wild-type mESCs at D0, and at ND D4, D6, and D8, was checked on Agilent Technologies-2100 Bioanalyzer, using an RNA nano-assay. All samples had RIN values of 9.8 or higher. Triplicate RNA-seq libraries were prepared (Illumina, San Diego, CA, USA TruSeq stranded mRNA protocol; www.illumina.com, accessed on 1 January 2020). Briefly, 200 ng of total RNA was purified using polyT-oligo-attached magnetic beads for ending with polyA-RNA. The polyA-tailed RNA was fragmented, and cDNA synthesized (SuperScript II, Invitrogen, Waltham, MA, USA, random primers, in the presence of Actinomycin D). cDNA fragments were end-repaired, purified (AMPureXP beads), and A-tailed using Klenow exo-enzyme and dATP. Paired-end adapters with dual index (Illumina) were ligated to the A-tailed cDNA fragments and purified (AMPureXP beads).

The resulting adapter-modified cDNAs were enriched by PCR (using Phusion polymerase) as follows: 30 s at 98 °C, 15 cycles of (10 s at 98 °C, 30 s at 60 °C, 30 s at 72 °C), 5 min at 72 °C. PCR products were purified (AMPureXP beads) and eluted in 30 µL resuspension buffer. One μL was loaded on an Agilent 2100 Bioanalyzer using a DNA-1000 assay to determine the concentration and for a quality check. Cluster generation was performed according to the Illumina TruSeq SR Rapid Cluster kit v2 Reagents Preparation Guide (www.illumina.com, accessed on 1 January 2020). After the hybridization of the sequencing primer, sequencing-by-synthesis was performed using a HiSeq-2500 with a single-read 50-cycle protocol followed by dual index sequencing. Illumina adapter sequences have been trimmed off the reads, which were subsequently mapped against the GRCm38 mouse reference (using HiSat2 version 2.1.0) [39]. Gene expression values were called using HTSeq-count version 0.9.1 [44] and Ensembl released 84 gene and transcript annotation. Sample QC and DEG analysis have been performed in the R environment for statistical computing (version 3.5.3, using DESeq2 version 1.22.1 [45] and Tidyverse version 1.2.1 (https://github.com/tidyverse;https://www.r-project.org/ from R Core Team, accessed on 1 January 2019).

### 2.11. Meta-Analysis, Pathways Enrichment, Gene Ontology, Function Analysis, and Gene to Disease Association

RNA-seq datasets (as DEG tables, from [23,36], were downloaded from GEO (https://www.ncbi.nlm.nih.gov/geo/, GSE35616, and GSE103003, respectively, accessed on 1 March 2019). Cross-referencing and visualization were performed in R using Tidyverse, VennDiagram, and pheatmap packages. The remaining analyses were performed with the StringDB package for R [46], while for Gene-to-Disease association Disgenet2R for R was used [47].

### 2.12. Zeb2 Short Hairpin (sh) RNA-Mediated Knock-Down

*Zeb2* knock-down (KD) was carried out by transfecting *Zeb2*-shRNAs into ESCs, at ND-D8. For this, the CAs were dissociated (see above), and single-cell suspensions were transfected using Amaxa Nucleofector II (using kit V, program A-33). Table 3 lists the shRNAs used in this study; the *Zeb2* target sequence is indicated in bold. In total 4 μg of shRNA was used for the transfection of 4.5 × 10^6^ cells. After transfection, the cells were plated in 5 mL of N2-medium on a poly-ornithine/laminin-coated 6-cm cell culture dish. Two hours post-transfection, the medium was refreshed, and 24 h after transfection was changed to N2-medium in the presence of puromycin (see above) for 48 h. The cells were then harvested, and KD efficiencies were examined using RT-qPCR. As a control, scrambled shRNA was used.

## 3. Results

### 3.1. Heterozygous Zeb2-V5 ESCs Differentiate as Wild-Type Cells

**Figure 1:**
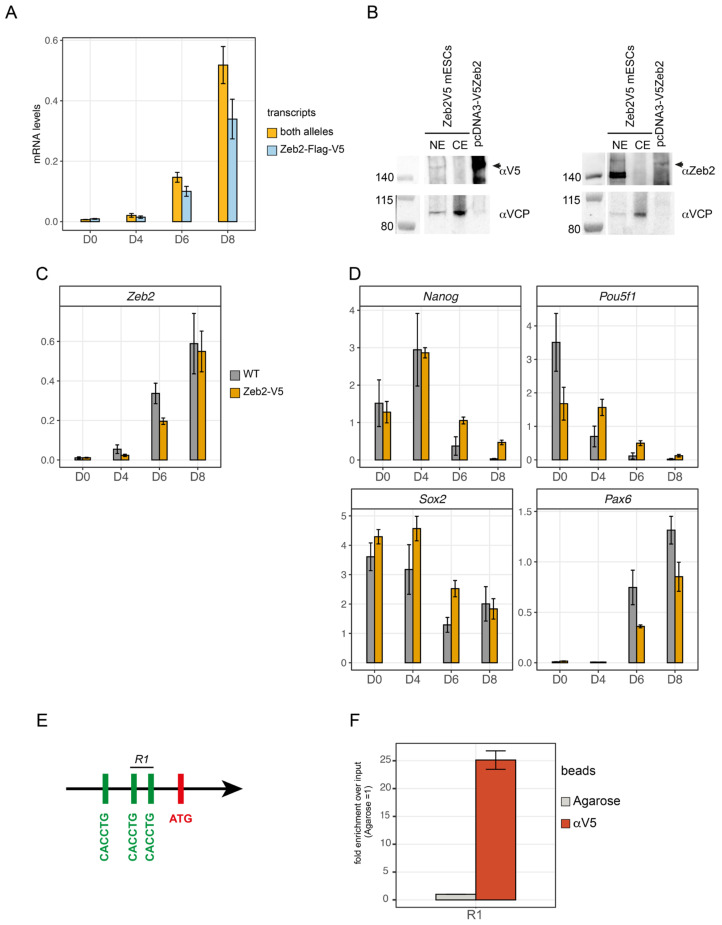
Characterization of the heterozygous Zeb2-Flag-V5 mESC line and ChIP-qPCR validation. (**A**) Allele-specific RT-qPCR using two sets of primers located either in exon7 (and therefore able to detect the whole Zeb2 mRNA produced by both alleles; orange bar) or located in exon9 and the V5-tag (thus recognizing specifically the knocked-in tagged allele; light blue bar); (**B**) Western blot analysis showing V5 epitope containing Zeb2 in ESC-derived NPCs (at D8 of neural differentiation, ND) in nuclear extracts (NE) and cytoplasmic extracts (CE). Membranes were blotted with anti-V5 antibody (left panel, αV5) or anti-Zeb2 antibody (right panel, αZeb2, [20]). As a control, a fraction of Zeb2-rich extract obtained from HeLa cells transfected with a pcDNA3-V5Zeb2 vector was also separated in the same gel; (**C**) Zeb2 mRNA levels in wild-type (WT, grey bar) and Zeb2-V5 (orange bar, clone 2BE3, indicated as Zeb2-V5) mESCs during ND, as determined by RT-qPCR; (**D**) RT-qPCR for ND, using marker genes: pluripotency marker genes *Nanog*, *Pou5f1* (*Oct4*) and *Sox2* are downregulated in Zeb2-V5 mESCs similarly to WT. The neuronal marker gene *Pax6* is also significantly upregulated during differentiation, such as in WT mESCs; (**E**) Scheme of the mouse *Cdh1* promoter showing the three E-boxes located upstream of the ATG start codon. Zeb2 binds specifically to only two of these, indicated as R1 [25]; (**F**) ChIP-qPCR showing enrichment for Zeb2-V5 binding to the R1 region of the *Cdh1* promoter. Agarose beads were used as negative control (in grey).

The addition of short epitope(s) at the N- or C-terminus, as well as activation/repression domains of heterologous transcription factors (TFs) at the Zeb2 C-terminus was previously shown not to interfere with Zeb2’s DNA-binding (as tested in *Xenopus* embryos [48], heterologous cells [49], mouse forebrain [36] and mESCs [25]). Here, we have used a CRISPR/Cas9 approach (see Materials and Methods) to insert an in-frame Flag-V5-tag encoding sequence in *Zeb2*-ex9 of mESCs (ESC clone 2BE3; Figure S1). Allele-specific RT-qPCR, using primers that amplify sequences between the ex9 and the V5-tag, showed mRNA expression from the tagged allele in ESC culture at day (D) 0, 4, 6, and 8 of ND as compared to the parental wild-type (WT) mESC line (Figure 1A).

Western blot analysis in nuclear extracts of ND-ESCs at D8 (thus NPCs, [25]) confirmed the presence of Zeb2 of expected molecular mass, using either anti-V5 (αV5) or anti-Zeb2 antibodies (Figure 1B). Both the Zeb2-V5 and wild-type (WT) ESCs were then also verified during ND differentiation for temporal expression of *Zeb2*, core pluripotency genes (*Pou5f1*, *Nanog*, both downregulated upon ND, and *Sox2*, also an NPC TF) and an acknowledged NPC marker (*Pax6)* (Figure 1C,D).

The untagged and tagged Zeb2 ESC lines displayed comparable expression dynamics of *Zeb2*, indicating that Zeb2-V5 NPCs at D8 of ND can be used for chromatin immunoprecipitation sequencing (ChIP-seq). Further confirmation came from the selective pull-down of Zeb2 on the known target *Cdh1*, using ChIP-qPCR. Zeb2 binds to two of three E-boxes in the mouse *Cdh1* promoter (Figure 1E), which it represses during epithelial-to-mesenchymal transition (EMT) [19,25]. A ±25-fold enrichment for Zeb2-V5 was obtained when probing this *Cdh1* region using anti-V5 antibody (αV5) conjugated beads compared to agarose beads as negative control (Figure 1F). Hence, Zeb2-V5 binds to known Zeb2 target sites, and the NPCs are suitable for endogenous mapping of the Zeb2 genome-wide binding sites (GWBS).

### 3.2. One-Third of 2432 Zeb2 DNA-Binding Sites Map Close to the Transcription Start Site of System-Relevant Expressed and Protein-Encoding Genes, Including the Zeb2 Gene Itself

**Figure 2:**
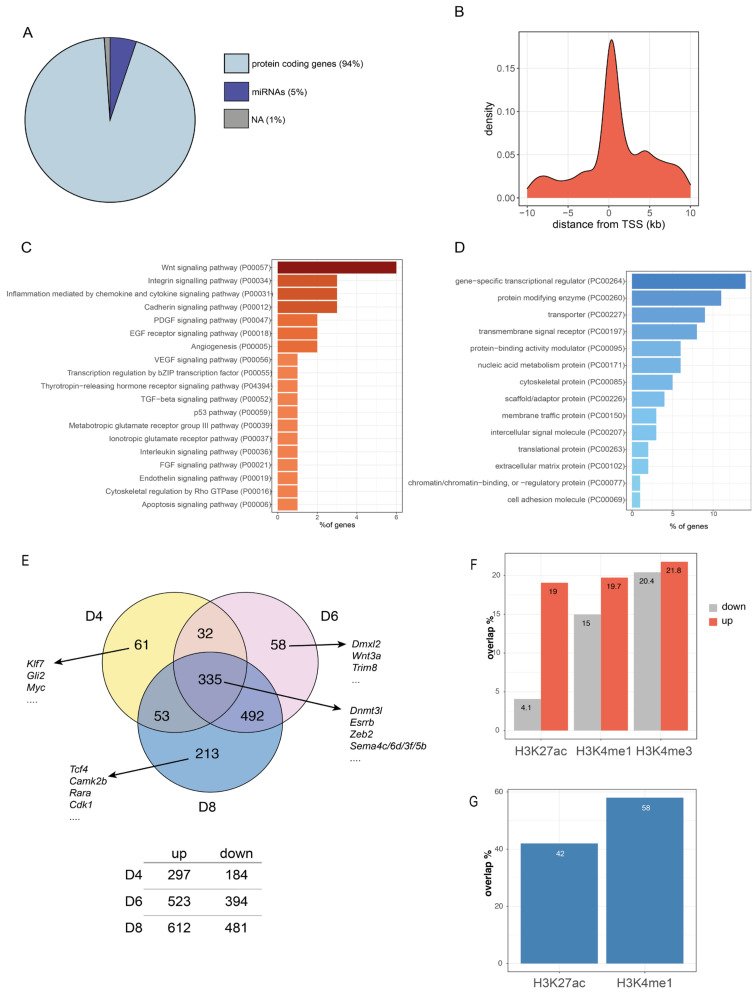
Zeb2-V5 protein is recruited at the TSS of transcriptional regulator encoding genes, predominantly those classified in Wnt signaling. (**A**) 2432 peaks were selected from our ChIP-seq data set (see Section 2). Of these, 94% are associated with protein-coding loci, 5% with miRNAs, and the remaining 1% map to regions without functional annotation (NA); (**B**) Frequency plot showing the binding of Zeb2-V5 at and around (−10 to +10 kb) the TSS; (**C**) The 2294 peaks map to 1952 protein-encoding genes, many of which operate in Wnt signaling (Table S1) or are (as shown in panel (**D**)) transcriptional regulators; (**E**) Of the 1952 protein-encoding genes, 1244 are differentially expressed during ND when compared to the undifferentiated state (D0). Of these 1244 genes, 335 are differentially expressed at all three time points of ND. We also list a few examples of DEGs uniquely expressed at one time point, as well as these that are shared among three time points; a full list is provided in Table S2; (**F**) Overlap of the Zeb2-bound regions with H3K27ac, H3K4me1, and H3K4me3 histone marks in the −10/+10 kb from the TSS of up or downregulated genes at D8 of mESCs differentiation; (**G**) Overlap of the Zeb2-bound regions outside the −10/+10 kb region from the TSS with histone marks.

αV5-precipitated samples from upscaled Zeb2-V5 NPCs were used for ChIP-seq (see materials and methods), followed by analysis with Galaxy Software [50]. Of the 2432 total significant peaks, 2294 peaks (94% of total) mapped to 1952 loci that encode proteins, while 125 peaks (5% of total) mapped to micro-RNA (miRNA) genes, and 1% to regions that lack annotation (NA, using ENSEMBL-GRCm38.99; Figure 2A; Table S1).

About 37.5% of all binding sites of Zeb2-V5 are located within −10/+10 kb of annotated transcription start sites (TSS, Figure 2B). Gene ontology (GO) pathway enrichment analysis of the aforementioned 1952 loci revealed binding of Zeb2 to classes of genes annotated to signaling by Wnt, integrin, chemokine/cytokine (predominantly as defined in inflammation) and cadherin, respectively, as well as to developmental signaling by EGF, VEGF, TGFβ, and FGF family pathways (Figure 2C). Among these 1952 loci, those for genes encoding transcription regulatory proteins, and post-translational modification as well as metabolic enzymes, are well-represented (Figure 2D).

In parallel, we applied bulk temporal RNA-seq of WT mESCs at D0 (undifferentiated), D4 (induction of ND), D6 (early NPCs), and D8 (NPCs) and checked the expression dynamics of the 1952 Zeb2-bound genes (from the D8 ChIP-seq sample). Among these, 1244 changed in steady-state transcription levels between D4-8 as compared to D0 (Figure 2E; log2FoldChange < −0.5 or >0.5 and *p*-value < 0.05; low-stringency analysis was opted to assess also small differences in mRNA of Zeb2-bound genes). Further, 335 of these genes, including Zeb2 itself, are commonly expressed between D4-6-8, but at different levels (for lists of all DEGs, see Table S2). Figure S2A depicts the D4, D6, and D8 transcriptomes of ND-mESCs, each compared to D0, with an indication of whether the genes are bound or not by Zeb2, as determined by our Zeb2-V5 ChIP-seq. At each of these respective time points, hence at different Zeb2 mRNA levels, about 11–14% of the up-/down-regulated DEGs are bound by Zeb2 (Figure S2B).

Among the Zeb2-bound genes that are normally down-regulated, *Dnmt3l* and *Esrrb* are present, suggesting that upregulation of *Zeb2* in ND-ESCs (D6 and D8) directly causes downregulation of these two genes accordingly (Figure S2C; Table S2). Zeb2 has been suggested as a direct repressor of *Dnmt3l* and *Esrrb*, facilitating the switch from self-renewal of ESCs to their exit from pluripotency, and promoting differentiation, since expression levels of all *Dnmt3* genes remained higher in *Zeb2*-knockout (KO) ND-ESCs [25]. However, these *Zeb2*-KO cells also convert very inefficiently into EpiLSCs and fail to exit from primed pluripotency. Importantly, among the Zeb2-binding genes whose mRNA levels increased during ND, *Zeb2* itself is also present (yellow dot, Figure S2C), indicating autoregulation. In fact, in this ND model, the highest recruitment of Zeb2-V5 in ChIP-seq data was mapped upstream of the TSS of Zeb2 (Table S1).

Out of the 1244 Zeb2-bound genes that significantly changed steady-state mRNA levels in our D4 to D8 transcriptome data sets, 213 are exclusive DEGs in D8 NPCs (Figure 2E and Figure S2D). Among these, *Tcf4* is bound by Zeb2 and increases in expression in NPCs (Figure 2E and Figure S2D). Tcf4 is a ubiquitous basic helix-loop-helix (bHLH) type TF that binds to E-boxes; its many isoforms [51,52] cooperate with cell-type specific bHLH TFs in heterodimers, which are active during CNS development [53,54] (for a review, see [55]). In oligodendrocyte precursors (OPCs), Tcf4 is essential for their subsequent differentiation. It dimerizes with the lineage-specific bHLH-TF Olig2, further promoting their differentiation and maturation [56], while Zeb2, together with upstream Olig1/2, is essential for myelinogenesis in the embryonic CNS [21]. Here, Zeb2 generates anti-BMP(-Smad)/anti-Wnt(-β-catenin) activities, which is crucial for CNS myelinogenesis by differentiation of OPCs. The regulatory action of Zeb2 on the *Tcf4* target gene, as found in mouse cells by our ChIP-seq, may underpin phenotypic similarities between MOWS and Pitt-Hopkins syndrome patients (PTHS, OMIM #610954; for a recent discussion, see [57]) the latter caused by mutations in *TCF4* [58], making us speculate that TCF4 may be deregulated in neural cells in MOWS.

### 3.3. Zeb2 Peaks Overlap with Active Enhancers and Promoters

To assess whether Zeb2-peaks are present in the regulatory regions of up or down-regulated genes from our transcriptome data, we cross-referenced the coordinates of the Zeb2 broad peaks within −10/+10 kb from the TSS with the mouse ChIP-seq datasets available in ENCODE for nervous systems (cerebellum, cortical plate, olfactory bulb, forebrain, midbrain, hindbrain, neural tube, and olfactory bulb, respectively). We found that, of these datasets from histone ChIP-seq available in ENCODE, the respective H3K27ac, H3K4me1, and H3K4me3 marks were overlapping with our ChIP-seq data (Figure 2F).

The H3K27ac signature strongly overlaps with the Zeb2 peaks in genes upregulated at D8 (19% in upregulated genes vs. 4% in downregulated genes). For H3K4me1 and H4K4me3 marks, no big difference in overlap between up and down-regulated genes was observed. While the H3K27ac mark is associated with active enhancers, H3K4me1 is associated with primed enhancers, and H3K4me3 is considered a “promoter” marker [59]. Taken together, these data suggest an activating role for Zeb2 here. Outside the −10/+10kb considered range, about 48% of the identified peaks had a 42% overlap with H3K27Ac and a 58% overlap with H3K4me1 histone marks (Figure 2G).

We then did motif enrichment analysis using UniBind (https://unibind.uio.no, 1 May 2022) for TFs that could bind the Zeb2-bound peaks or could do so in proximity to up or down-regulated genes at D8 of mESCs differentiation (Figure S3A,B). In those peaks close to the TSS of upregulated genes, binding motifs for Sox2, Gata2, and Tcf3 are very abundant. These TFs are known to function during NPC or ND. For example, Sox2 is an acknowledged marker for neurogenesis [60]. It has been demonstrated that Zeb2 is needed to elicit anti-Sox2 activities in (re)myelination by adult Schwann cells in the PNS, needed for normal progression of commitment, differentiation, and maturation in this glial cell lineage [21,24,61]. Tcf3 (also known as E2A) plays a role in stem cell self-renewal [62], but is also important during neural fate commitment and possibly repressing Nodal signaling during ND [63]. Gata2 has been associated with negative regulation of proliferation in NPCs and, as a result, further differentiation of these cells [64]. However, how Zeb2 acts upon or together with these TFs during NPC differentiation is not fully known yet.

In the peaks close to the TSS of downregulated genes there is a prevalence for CTCF, Fos, Myc, and Stat5a binding. CTCF is of interest because it acts as a link between 3D genome architecture and gene expression regulation. During NPC differentiation however, it was observed that 40% of the NPC-specific DNA loops were not CTCF-dependent, whereas, in other cell-state specific loops, this was only 10%, indicating a less important role for CTCF in the regulation of NPC differentiation compared to other cell lineages [65]. This might indicate an interesting role for Zeb2 in binding and possibly regulating CTCF mRNA levels during NPC differentiation, supporting the subsequent activation of NPC-specific genes arising from e.g., repressing CTCF. Also here, more studies are required to get more insights into the cooperativity or counteracting actions of Zeb2 with candidate TFs in the regulation of the candidate Zeb2 targets.

### 3.4. Meta-Analysis of Identified Binding Sites and Perturbed-Zeb2 RNA-Seq Data Reveal Overlapping Zeb2 Target Genes

**Figure 3:**
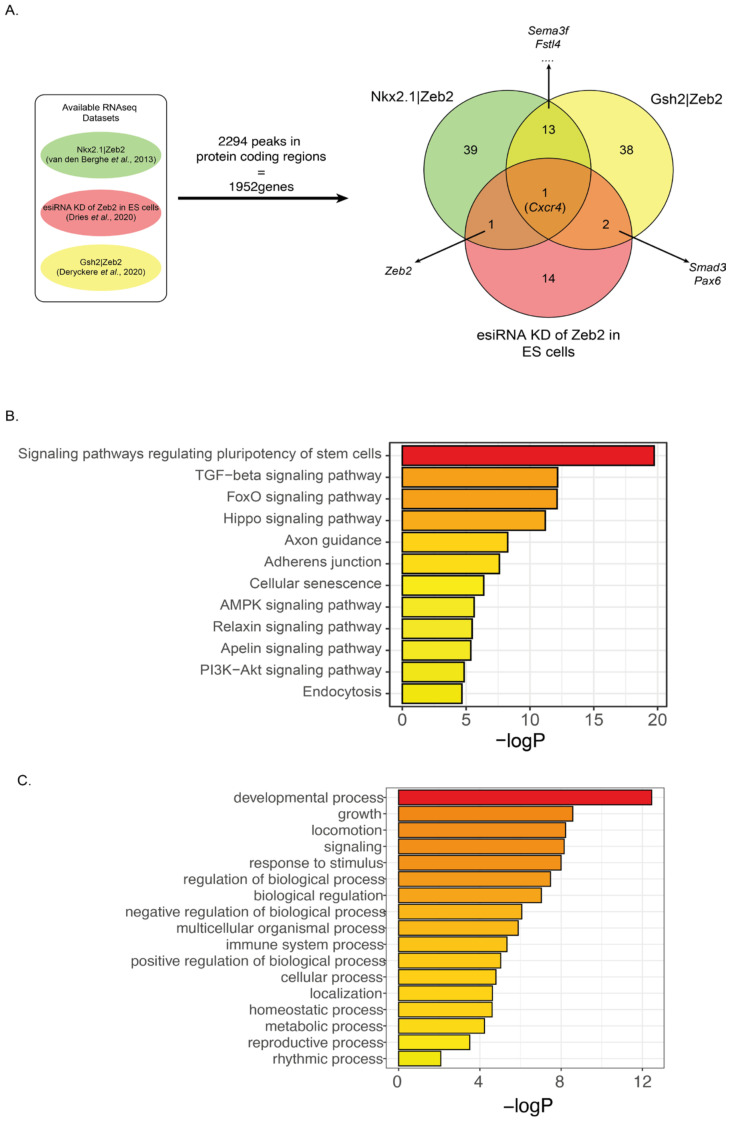
Schematic representation of the meta-analysis of Zeb2−bound genes versus RNA−seq datasets. (**A**) 108 genes bound by Zeb2 are also differentially expressed in the three data sets from other studies in mouse models and ESCs (see main text for details, [23,36,37]). *Cxcr4* is the only DEG bound by Zeb2 and common among the three data sets (for discussion, see main text); (**B**) These 108 genes mainly map to signaling pathways regulating stem cell pluripotency, and effects of TGFβ family, FoxO, and Hippo signaling/activity; (**C**) The 108 genes cluster as important regulators of developmental processes, locomotion, and signaling.

We performed a meta-analysis of three published transcriptome data sets from control and *Zeb2*-KO mice: sorted E14.5 mouse ventral forebrain interneurons (Nkx2.1-Cre driven *Zeb2*-KO; [36]) and sorted (at P2) progenitors of the ventricular-subventricular zone (V-SVZ), an adult neurogenic niche in the forebrain (Gsh2-Cre; [23,36]). In addition, we used high-throughput RT-qPCR data generated on a Fluidigm platform and obtained after esiRNA-based knockdown (KD) of Zeb2, as part of a systems-biology study in ND-mESCs [37] (with the Zeb2 KD data subset kindly provided by R. Dries, Boston University). From these respective datasets, the DEGs upon the Zeb2 perturbations (*p*-value < 0.05; log2FoldChange < −1 and >1) were filtered. This identified 108 genes in total, and these depend on normal Zeb2 levels for their (i) downregulation/repression (if directly by Zeb2, as a repressor) or (ii) other genes that depend on Zeb2 for their upregulation/activation (if directly by Zeb2, as an activator) (for Zeb2 as dual TF [29,30,66]. In parallel, the 2294 Zeb2-V5 sites mapping to the 1952 protein-encoding genes were filtered from the complete ChIP-seq dataset and then used as references for the RNA data sets (Figure 3A).

Thus, this cross-referencing identified 108 protein-encoding genes among the three transcriptomic data sets and the ChIP-seq data set (Figure 3A, Table S3). Figure S4 shows a heatmap of the changes in mRNA levels of these 108 genes during ND of wild-type ESCs and their correlation with the analyzed datasets. Noteworthy, only *Cxcr4* was common to all RNA data sets. This is likely due to the fact that two RNA-seq sets are generated in different brain/neuron cell-type in vivo mouse models, while the other steady-state RNA level data documented the effects of Zeb2-KD on mRNA levels of (only 96 in total) TGFβ/BMP-system components [37], so the timing does not completely overlap with our ChIP-seq dataset. However, Cxcr4 and its ligand Cxcl12/Sdf-1 are crucial for migration of interneurons from the ventral forebrain to the neocortex [67,68], processes co-controlled by Zeb2 as shown in cell-type specific KO mice [36]. Furthermore, the identified 108 genes are involved in the regulation of stem cell pluripotency, signaling by TGFβ, FoxO, and Hippo, and in axon guidance. Taken together these data further confirm the pivotal role of Zeb2 in these processes. Further Gene Ontology (GO) analysis reveals that these 108 genes cluster as important regulators of developmental processes, cell locomotion, and signaling (Figure 3C). These processes are affected in human conditions following *ZEB2* heterozygosity, as in the case of MOWS.

### 3.5. Zeb2 Directly Controls TGFβ/BMP-System Component and Neuronal Differentiation/Migration Genes

**Figure 4:**
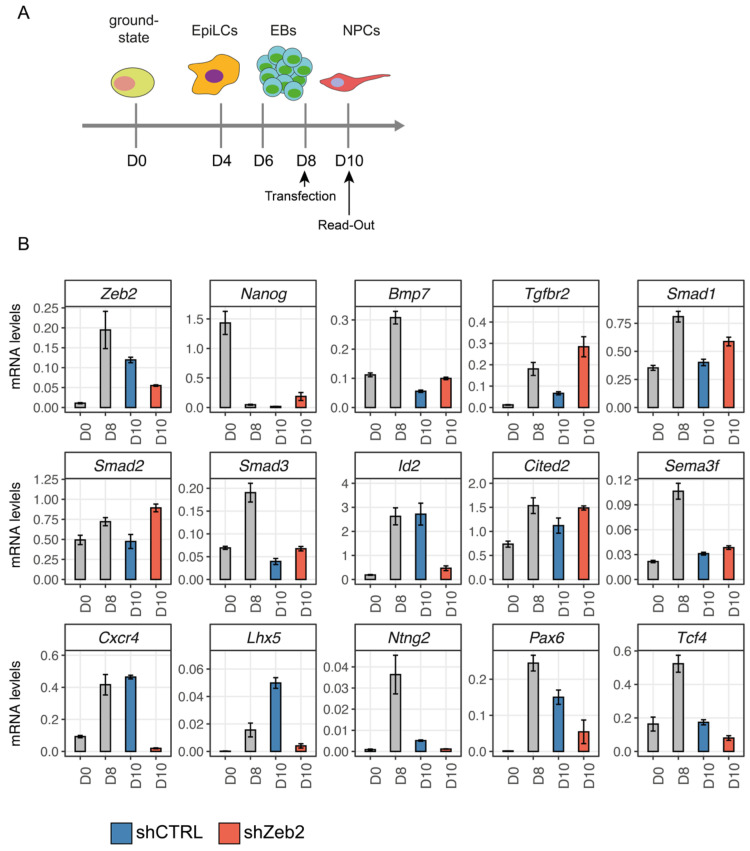
shRNA-mediated KD of Zeb2 discriminates between primary and secondary target genes. (**A**) Schematic overview of the shRNA transfection targeting Zeb2 (shZeb2) and read-out of the effect. Cellular aggregates at D8 of ND are dissociated and transfected with shZeb2 or against a scrambled, control sequence (shCTRL). Read-out is done two days after the start of shRNA addition. The list of shRNAs is given in Table 3. (**B**) RT-qPCR measurements: Zeb2 levels after KD were reduced to 40–50% of their normal level (shZeb2, orange bars) compared to shCTRL (blue bars). *Bmp7*, *Cited2*, *Nanog*, *Sema3f*, *Smad1*, *Smad2*, *Smad3*, and *Tgfbr2* were upregulated following Zeb2 KD, whereas genes encoding for neuronal specification and migration (*Cxcr4*, *Lhx5*, *Ntng2*, *Pax6*, and *Tcf4*) were downregulated.

We then validated 14 out of the 108 cross-referenced target genes, selected based on either being already known as a target of Zeb2 (*Nanog*, [25]), or as TGFβ/BMP-system component (*Bmp7*, *Tgfbr2*, *Smad1*, *Smad2*, *Smad3*, *Id2*, *Cited2*), or having a crucial role in neurogenesis and neuronal maturation (*Sema3f*, *Cxcr4*, *Lhx5*, *Ntng2*, *Pax6*, *Tcf4*; their mRNA levels in ND-ESCs are highlighted in the heatmap in Figure S4). Because *Zeb2*-KO ESCs do not exit from primed pluripotency and thus cannot differentiate [25], we validated our findings using shRNA-mediated Zeb2-KD at ND-D8 and analyzed these aforementioned 14 genes two days later (D10 NPCs) (Figure 4A). At this read-out time point, >50% reduction of Zeb2 mRNA expression was obtained (Figure 4B).

Zeb2-KD resulted in reduced mRNA levels of *Cxcr4*, *Ntng2*, and *Pax6* (Figure 4B), genes that are each involved in neuron specification and migration [69,70]. Zeb2-KD also caused down-regulation of *Lhx5*, involved in the differentiation of interneurons, including cytoskeletal rearrangements during dendritogenesis [71], and of *Tcf4*, which acts in neurogenesis [54,55]. Sema3f is a cue for axon outgrowth and neuron migration guidance, and its gene was slightly upregulated (Figure 4B). These results confirm the regulation by Zeb2 of its direct targets in later phases of neuronal differentiation/migration.

The expression of Nanog, the promoter of which binds Zeb2 as a repressor [25], was increased in the Zeb2-KD cells (Figure 4B). Zeb2-KD caused increased mRNA of *Bmp7*, *Tgfbr2*, *Smad1*, *Smad2*, *Smad3*, and *Cited2* (Figure 4B), fitting with the normal levels of Zeb2 that mount anti-TGFβ/BMP family effects [29]. In contrast, *Id2* is strongly downregulated in shZeb2-treated ESCs (Figure 4B). *Id2* is normally activated by BMP-Smads and, together with other Id proteins (Id1, Id3, and Id4), inhibits cell differentiation, e.g., Zeb2 represses *Id2* in immune cells to promote differentiation [22]. However, *Id2* as well as other *Id* genes [72,73,74] is, such as *Zeb2* [20], also expressed in the developing forebrain.

Taken together, these data suggest an active and direct role for Zeb2 in repressing genes regulating stem cell pluripotency as well as a number of TGFβ/BMP-system components (*Bmp7*, *Smad1/2/3*), but also in activating genes during neurogenesis (*Cxcr4*, *Ntng2*, *Lhx5*).

### 3.6. Zeb2 Potentiates Its Own Gene Expression, Which Is Crucial for Proper Control of Some of Its Direct Target Genes

**Figure 5:**
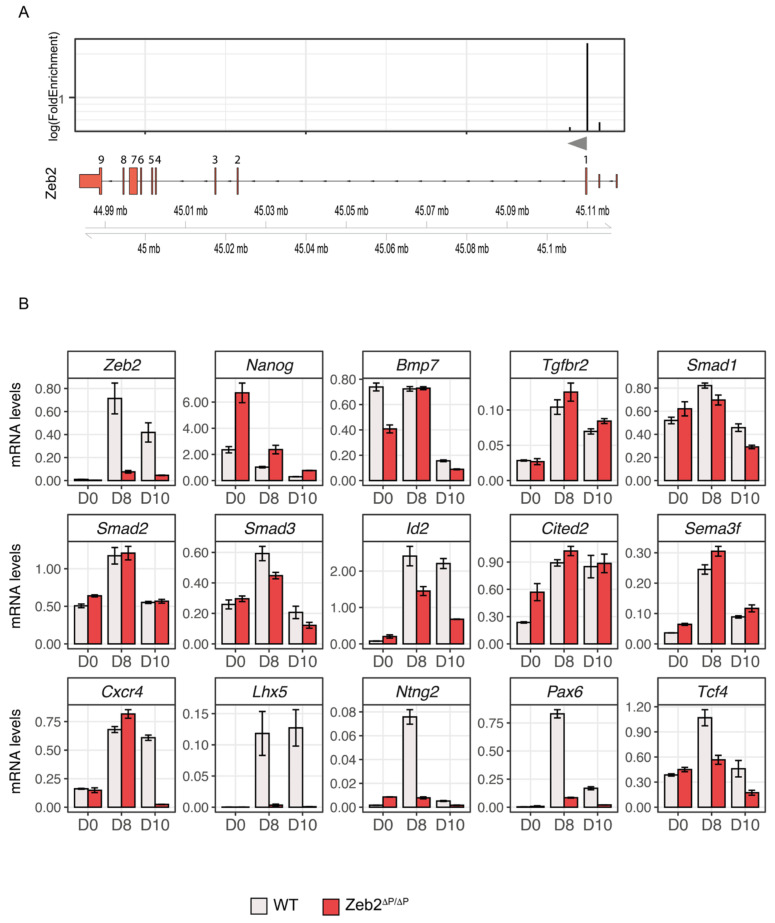
Deletion of the Zeb2-binding autoregulatory site (chr2:45109746-45110421) impairs Zeb2 mRNA levels and neuronal markers. (**A**) Schematic overview of the log (FoldEnrichment) of the peaks identified by ChIP-seq located in the mouse *Zeb2* locus, and localization on top of *Zeb2* intron/exon structure. The highest peak is located 232 bp upstream of the first translated exon. The grey arrow indicates the TSS of *Zeb2*; (**B**) RT-qPCR: Zeb2 mRNA levels are strongly reduced in the *Zeb2^ΔP/ΔP^* clone. For the target genes validated with shRNA (see also Figure 4), most, but not all of the genes found to be affected following Zeb2 KD are also deregulated in *Zeb2^ΔP/ΔP^* mESCs, in particular Id2 and the neuronal markers *Cxcr4*, *Lhx5*, *Ntng2*, *Pax6*, and *Tcf4*.

Strikingly, in our ChIP-seq dataset, the peak with the highest enrichment (~200-fold) mapped 232 bp upstream of the *Zeb2* TSS (Figure 5A, Table S1). For further functional studies of this site, we deleted the encompassing region (chr2:45109746-45110421) using CRISPR/Cas9 in wild-type mESCs, thereby obtaining *Zeb2^ΔP/ΔP^* ESCs (Figure S5 see Section 2). Figure 5B shows that the Zeb2 mRNA levels in the homozygous ΔP clone stayed strikingly low during ND, already from D8 onwards, compared to control cells. We then used these *Zeb2^ΔP/Δ^*^P^ mESCs to read out the same genes that depend on intact Zeb2 levels and are Zeb2 ChIP+ (see Figure 4B). Levels of Zeb2 mRNA stayed abnormally low at D10 in *Zeb2^ΔP/ΔP^* ND-mESCs, whereas *Nanog* was still expressed and remained higher than in control WT cells (Figure 5B). Hence, Zeb2 levels are critical, albeit to a different degree for sets of genes. The latter include neuronal-relevant genes such as *Cxcr4*, *Lhx5*, *Ntng2*, *Pax6*, and *Tcf4* (Figure 5B). Among the TGFβ/BMP-system components (see Figure 4) we observed a limited reduction of *Bmp7*, *Smad1*, and *Smad3*, whereas *Tgfbr2*, *Smad2*, *Cited2*, and *Sema3f* expression was not affected in *Zeb2^ΔP/ΔP^* ND-mESCs. Based on these results, we speculate that Zeb2, the precise amounts of Zeb2, and in a critical stage also its autoregulation, are crucial in discriminating genes where Zeb2 plays the aforementioned primary, active role (as for *Cxcr4*, *Lhx5*, *Ntng2*, etc.). For these genes, ~50% reduction or mutation of the autoregulatory binding sequence is sufficient to strongly deregulate them, but other genes’ expression is either not or just slightly affected (*Sema3f*, *Smad2*, *Cited2* vs. *Bmp7*, *Tgfbr2*, *Smad1*, *Smad3*).

Zeb2 also binds phospho(p)-Smads [4,21,23,29]. Therefore, we also scanned the Zeb2 ChIP+ direct target genes deregulated upon Zeb2-KD and/or in the *Zeb2^ΔP/ΔP^* cells during ND (i.e., without Smad activation) for the presence of (i) the Zeb half-sites CACCT(G) [3] and (ii) candidate p-Smad binding and responsive genes (using GTC(^T^/_G_)CT(^T^/_G_)(^A^/_C_)GCC for p-Smad1/Smad5, GTCTAGAC for p-Smad2/3) and (iii) the co-Smad Smad4 (C(^C^/_T_)AGAC), using the Jaspar database (for a review on Smad target sites, see [75]; see also Section 2). Figure S6 shows the distribution of such identified Zeb and Smad-binding motifs (threshold score > 85%) in those genes strongly affected by Zeb2-KD and/or in *Zeb2^ΔP/ΔP^* cells. Interestingly, in the regions where Zeb2 binds close to the TSS (*Zeb2*, *Ntng2*, *Lhx5*, *Nanog*), the p-Smad and Smad4 binding elements are sometimes present in very close proximity to the ChIP+ Zeb2-bound E-box, indicating a possible cross-talk between receptor-activated Smads and Zeb2 in regulating target genes.

### 3.7. Extrapolation of Zeb2 ChIP-Seq Data to Cell-Based Clinical Manifestation of MOWS

Despite the unprecedented nature of our Zeb2 ChIP-seq data obtained in ESC-derived NPCs in this study, an ideal extrapolation to MOWS, and in particular its clinical manifestation, is not straightforward and remains speculative (see Section 4). In this respect, the selected three experimental models of Zeb2 perturbation (including the two Zeb2-cKO models with cellular phenotypes that reveal underlying defects in MOWS [23,36]) present a more suitable intermediate hold, considering our list of 108 target genes for Zeb2. These options keep in mind the molecular and cellular consequences (e.g., gene expression profiles, cell differentiation states) of Zeb2 deficiency in these mouse models (for a recent review of most mouse models, see [30]). In addition, we considered deficiencies (with a focus on neurodevelopmental defects) in MOWS patients as documented by clinicians [8,9,10,11,12,13,14,15,16,17,18]. The realistic options in trying to correlate Zeb2 ChIP-seq and Zeb2-perturbation RNA-seq data with MOWS in the clinic are presently three-fold. They each consider the aberrant expression of putative Zeb2-dependent direct target genes in (i) tissues/cells from MOWS patients, including MOWS iPSCs and derived neurons. However, this work in the MOWS field is only starting now. Therefore, we did this (ii) first for each of the 108 identified genes (Table S4), and then (iii) repeated this exercise for our entire list of genes to which Zeb2 binding sites were assigned (Table S1). Again, in both exercises, we tried to relate genes to clinical consequences in MOWS.

The Zeb2-dependent direct target genes within the list of 108 genes from this study were first grouped based on cellular functions that may relate to aspects of MOWS, and within these groups, relevant gene candidates for further investigation in extra cell systems in the future were then proposed, such as MOWS iPSCs that have been driven into neural differentiation and possibly neuron subtypes (option (i) above). Table S4 shows such genes for (calcium) ion-binding and channels (with *Syt13*, *Pcdh9*, *Nalcn*, and *Kcnj6* as candidate relevant genes); or operate in synapse biology (with again *Pcdh9*); axon outgrowth, guidance, and connectivity (with many genes, including *Epha5* and *Sema3f*); neuron subtype specification (including *Pax6*, *Klf7*, again *Efna5*, and *Lhx5*, *Hmx2*); motoric capabilities (with again *Nalcn* and *Lhx5*); and genes related to neurodevelopmental disorders and behavior (such as *Npas1*, *Cacng5*, *Foxp1*, *Wdr62*, and again *Kcnj6*).

Interestingly, some of these candidate genes for priority inclusion in future MOWS cell-based studies also emanated from independently overlooking all mapped GWBS for Zeb2 from this study (in Table S1) with an eye for anticipated MOWS cell biological defects, focusing mainly on neural and glial cells. These genes include *Bcl11* (developmental intellectual disorders, agenesis of corpus callosum), *Caln1*, *Efna5*, *Galnt5/6*, *Gng4/7*, *Isl1* (a TF that regulates expression of *Slit* and *Robo* genes), *Klf2/7/14*, *Nalcn*, *Pax6*, *Pcdh9/20*, *Pipox*, *Pou3f/4f* members (one also known as *Brn3a*), *Ror2*, *Sema3*, *Slc14a2*, *Sox1*, *Tcf4* (see [57] for a detailed discussion), *Tubb3/6*, and of course *Zeb2*. From Table S1, we would also prioritize genes for adhesion G-coupled receptors (e.g., *Adgre5*, *Adgrl2*), adherens junctions (*Ajap1*, *Frmd4a*, *Jam3*), several *Cdh* genes, genes involved in Wnt (*Axin2*, *Kremen1*) or BMP signaling (*Rgmb*, *Ror2*), sulfotransferase-encoding genes (*Chst2/7*, *Hs3st3a1*, *Ndst1*), *Camkk1* (for MOWS patients have CAMK deficiency), chemokine receptor genes (*Ccr1/7*), *Ddx10/18*, *Efhb*, *Fgf14*, *Lrrc4c* (encoding a binding partner of long-range guidance cue Netrin G1), *Nrn1* (encoding a neuritin, involved in neuronal plasticity), *Pitx2*, *Plk2* (encoding a kinase that links to epilepsy), *Prex1*, *Prox1*, *Robo2*, *Six2*, *Snai3*, *Sox5*, *Tenm3* (for proper connectivity in the nervous system), and Tox3 (chromatin bending).

## 4. Discussion

We report for the first time the endogenous genome-wide binding sites (GWBS) for Zeb2, in ESC-derived NPCs. In previous work, we have used ESCs established from *Zeb2^Δex7/Δex7^*-KO [76] pre-implantation embryos and, for rescue purposes, such KO ESCs cells in which Flag_3_-Strep-Zeb2 was produced from (a Cre-controllable) *Rosa26* locus [25]. The latter cells are different from the mESCs that were established here, since *Zeb2* is not subjected to its normal temporal regulation during cell differentiation, in contrast to the Flag-V5 mouse (m) ESCs obtained here.

Precise dosage of Zeb2 is however a critical factor in vivo (for a recent discussion, see [30]). This is concluded from transgenic Zeb2 cDNA-based rescues in *Zeb2*-KO ESCs and similar genetic rescues in Zeb2-mutant cells in mice, which via heterozygous/homozygous combinations create an elegant and large panel of Zeb2 mRNA levels (in interneurons [36], NK cells [77], ESCs [25]). Another illustration of the relevance of fine-tuned control of Zeb2 levels are miRs that target Zeb2, and lncRNAs that regulate these miRs [78,79,80,81], with Zeb2 in its turn also controlling some of its own miR-encoding genes or clusters [82,83,84]. In our ChIP-seq, we find 125 peaks (~5% of the total) that correspond to the TSSs of 98 miR genes (Table S1). Among these miR genes, Zeb2 binds to loci encoding miR-144, miR-148a, miR-9, and miR-153, known to target Zeb2 in the context of e.g., tumor EMT and tumor progression [85,86,87,88]. We recently added the identification, in human iPSCs subjected to ND, of ZEB2 distant (~600 kb upstream) enhancers, which act through DNA-looping to the ZEB2 promoter-proximal region [89]. In addition, we have documented dynamic expression patterns of Zeb2 in early embryos [27,28,33,90,91,92]. We have also shown that cDNA-based expression of various tag-Zeb2 proteins is compatible with functional embryology-type and action mechanism studies [25,48,49]. Importantly, our *Zeb2-V5* allele steers normal production of tag-Zeb2 from its endogenous locus.

Only two studies present ZEB2 ChIP-seq data in human cells, i.e., SNU398 hepatocellular carcinoma and K562 erythroleukemia cells, respectively. In K562 cells ZEB2 binds to the promoters of *NR4A2*, *NEUROG2*, and *PITX3*, expressed in midbrain dopaminergic neurons, wherein—in mice—Zeb2 negatively regulates axon growth and target innervation [93]. In SNU398 cells, ZEB2 represses *GALNT3*, which is normally expressed in epithelial cells. This repression coincides with the acquisition of a mesenchymal phenotype, linking ZEB2 here again to an EMT-like process. These two valuable studies also present limitations. The use of cancer cell lines of a genomica unstable nature may create possible bias in ChIP-seq, and in any case, they overproduce ZEB2. Our ChIP-seq identifies >2400 peaks for Zeb2-V5 in mESCs at ND-D8; 37.5% of the Zeb2 sites map close to TSSs (when defined as −10/+10 kb). Most of these genes function in growth factor or cytokine signaling and/or encode transcriptional regulators. The latter suggests that Zeb2 orchestrates other cooperating TFs driving the transcriptomic signature of NPCs. The regulation of Wnt signaling by Zeb2 is in line with observations that inhibition of the Wnt-βcatenin pathway suppresses ND in vitro and in vivo and that Wnt (and Zeb2)-controlled *Tcf4* expression promotes neurogenesis and is required for normal brain development [94,95,96,97,98].

The 2294 Zeb2 peaks map to 1952 protein-coding genes, of which 1244 are DEGs in NDmESCs. Strikingly, the strongest enrichment of Zeb2 occurs on the *Zeb2* promoter itself, leading to the identification of a novel self-regulatory mechanism where Zeb2 binds upstream of its TSS to maintain its levels sufficiently high, at least during ND. While this autoregulation needs further investigation in Zeb2-dependent differentiation and/or maturation of other cell types (e.g., in cKO mouse models or in ND-iPSCs derived from appropriate MOWS patient cells), we propose that lower Zeb2 levels might compromise this autoregulatory loop. Deletion of the autoregulatory site from both Zeb2 alleles (in the *ΔP/ΔP* cells), results in a significant decrease of Zeb2 mRNA levels but *Zeb2* is still partially expressed, and these cells can still exit from pluripotency and differentiate. A number of genes, which are mainly linked to neuron maturation, are affected in *Zeb2^ΔP/ΔP^* ESCs, whereas TGFβ/BMP-system component genes are not deregulated. Zeb2 dosage might thus underlie this difference in regulating its direct, ChIP+ genes in our ESCs. Zeb2 might be key to maintaining expression of neuronal genes, while for TGFβ/BMP system genes Zeb2 may cooperate with other TFs (including p-Smads) or DNA-modifying enzymes to regulate the expression of target genes.

Zeb2 binds to TGFβ/BMP family receptor-activated phospho-Smads (pSmads), and several studies indicate its negative regulation of BMP-Smad activation of specific target genes, although Zeb2 also has Smad-independent functions [23,29]. BMP-pSmads bind to GGCGCC with high affinity [99]. Morikawa and co-workers [100] have confirmed these results using ChIP-seq, and also identified a lower-affinity (so, higher BMP-doses required) BMP-Smad element (GGAGCC). To achieve full responsiveness, it was proposed that the GG(^A^/_C_)GCC element needs to be coupled with a Smad4 site, optimally located 5 bp away [100]. We find that in primary targets affected by varying levels of Zeb2, E-boxes are located close to Smad-binding motifs. However, whether Zeb2 and Smads are co-present in target regions requires further experiments, such as ChIP-on-chip assays, and (non-neural) differentiation protocols (involving stimulation of the cells by addition of BMP and/or Nodal). However, these studies may be further complicated because of the post-translational modification status of Zeb2, nuclear p-Smads, and/or Smad4 [101,102,103,104].

The 1093 Zeb2-binding DEGs at D8 are also striking. When we performed a gene-to-disease association using the human orthologues of these D8-DEGs, we found a clear association with several disorders (Figure S6). These include neurodevelopmental, mental, and ocular defects, which occur in MOWS. Altogether, our data may provide novel insights into MOWS due to suboptimal ZEB2 amounts in patients, and from now includes a gene autoregulation aspect, as well as *ZEB2* as a putative modifier gene for many other congenital disorders.

Several *Zeb2*-cKO mouse models have been generated, and many bulk RNA-seq data are available, from which here we selected two such data sets [23,36,105]. In addition, similar data were obtained from cultured mESCs, either *Zeb2*-KO cells (25) or cells submitted to ND wherein e.g., Zeb2-KD was performed [37]. We could not include *Zeb2*-KO mESCs in these comparisons, for they convert dramatically less efficiently to EpiLSCs and they fail to differentiate beyond this EpiLC state [25]. The meta-analysis of the three different used data sets, overlaid with the 1952 Zeb2-V5 ChIP+ loci/genes, show therefore a limited number of common targets, *Cxcr4* being the only one common to all data sets. Throughout all three different datasets, we could narrow down the target gene collection to 108 Zeb2-bound genes in total. However, interestingly, these 108 genes enrich GO terms such as pluripotency of stem cells, signaling by TGFβ and Wnt, cell fate commitment, and neuron differentiation, all processes wherein Zeb2 plays a crucial role.

Out of these 108 genes, we selected 14 covering TGFβ/BMP signaling, pluripotency, neuron migration, and differentiation/maturation, and checked their levels two days after Zeb2-KD at ND-D8. Most of these 14 genes relevant to NPC status were shown to be critically dependent on intact levels of Zeb2. They may help to explain why the defects caused by MOWS are observed later after birth and why (the few) missense mutations in MOWS (besides the more abundant significant deletions) present with milder syndromic manifestation. Both the cross-reference of Zeb2-ChIP+ genes with the transcriptome of ND-ESCs and the meta-analysis identify a number of common genes, such as *Bmp7*, *Tgfbr2*, *Tcf4*, *Smad1/2/3*, and *Sema3f* (Table S3; Figure S4), making Zeb2 a likely direct regulator of these genes. It is also intriguing that Zeb2 is recruited to and controlling *Tcf4* at D8, and that *Tcf4* is deregulated upon Zeb2-KD (using esiRNA [37], and here shRNA). Mutations in *TCF4* cause PTHS, a rare neurodevelopmental disorder with some defects overlapping with MOWS (for an extensive discussion, see [57]). The binding of Zeb2 to *Tcf4* opens new attractive roads to further investigate the crosstalk between these two TFs and their role in regulating crucial aspects of neurodevelopment.

Despite the novelty of our mapped Zeb2 GWBS, the extrapolation of this work to the clinical manifestation of MOWS itself is interesting but remains speculative. First, all Zeb2-dependent genes whose mRNA levels change upon Zeb2 perturbation in vivo, and that are relevant to explain aspects of MOWS (for example, neurodevelopmental deficiencies; see [30]), have been identified in homozygous *Zeb2*-KO cell types from mouse models, whereas MOWS in humans is caused by heterozygous *ZEB2* mutations [12,13,14,15,16,30]. At present, we do not know what such heterozygous MOWS mutation means with regard to *ZEB2* autoregulation in patient cells. However, the *Zeb2*-KO mouse models, if ideally including phenotyping by RNA-seq, have allowed for the proposal of cellular mechanisms underlying major MOWS-related defects in humans. Examples would be the neurocristopathies that impact craniofacial and enteric nervous system development, leading to Hirschsprung disease in (most) MOWS patients [9,10,16]. Another defect, intriguingly but largely clinically unexplored, concerns the development of dorsal root ganglia, relating to possible pain deficiencies in MOWS patients. Key examples may be nervous system deficiencies, such as those found in the timing of neuro-/gliogenesis in corticogenesis in KO mice, relating to intellectual disability in the patients [20,23,36], and those in guided migration and connectivity of forebrain interneurons in KO mice, relating to seizures and epilepsy in MOWS patients [36]. Confirmatory gene expression profiling studies have, to our knowledge, not been carried out on tissues/cells from MOWS patients. Furthermore, this approach would be restricted to cells from post-natal and adult tissues and would focus on fully differentiated cells, e.g., mature neurons. In reverse, our ESC-to-NPC-based cellular model has not been explored yet towards differentiation of specific neuron subtypes, and their maturation in vitro. Last but not least, the establishment of iPSCs from MOWS patients directly or as the result of *ZEB2* (e.g., heterozygous inactivation) gene-editing in wild-type iPSCs is still in a very early phase. Such iPSCs would even be more ideal if the single remaining, intact wild-type *ZEB2* allele would also be epitope-tagged—as carried out here in mESCs—for repeating the ChIP-seq experiments upon neural differentiation. With these careful considerations in mind, and in an attempt to correlate this Zeb2 ChIP-seq work with the clinical manifestation of MOWS, we nonetheless tentatively propose a number of candidate relevant genes for further investigation that might be relevant to MOWS as it presents in the clinic (see Results Section 3.7). These speculations do not yet include other crucial Zeb2 functions—as documented mainly in mouse models [30], such as in (re)myelination [21,24,61], diverse cell types of the immune system [22,34,77], and perhaps the formation of the immune synapse, and fibrosis [106]. Some of these novel phenotypes in mice prompt many clinicians to start longitudinal follow-up in MOWS patients also from these perspectives.

## Figures and Tables

**Table 1:** List of primers used in the study.

Primer Name	Sense/Antisense	Sequence (5′ → 3′)	Application
FlV5mZeb2Ex9_Fwd	Sense	GGCTTACCTGCAGAGCATCA	genotyping
FlV5mZeb2Ex9_Rev	Antisense	CTCCATCTAACTCTGTCTTGGC	genotyping
FlV5_Fwd	Sense	CTACTCGCAGCACATGAATC	genotyping
FlV5_Rev	Antisense	GAGAGGGTTAGGGATAGGC	genotyping
ΔZP_P1_Fwd	Sense	GTCAGTCCGTCCCCAGGTTT	genotyping
ΔZP_P2_Rev	Antisense	GGCATGCTAGCTGGGCTGGT	genotyping
LN249_Fwd	Sense	GGAGCAAACTGAACAAAACCTCGCC	genotyping
LN249_Rev	Antisense	GGCGAGGTTTTGTTCAGTTTGCTCC	genotyping
LN209_Fwd	Sense	AGCGGATCAGATGGCAGTTCGCATG	genotyping
LN209_Rev	Antisense	CATGCGAACTGCCATCTGATCCGCT	genotyping
Zeb2_Fwd	Sense	CAATGCAGCACTTAGGTGTA	qPCR
Zeb2_Rev	Antisense	TTGCCTAGAAACCGTATTGT	qPCR
Zeb2V5_Fwd	Sense	GAAACGATACGGGATGAGGA	qPCR
Zeb2V5_Rev	Antisense	AGGAGAGGGTTAGGGATAGG	qPCR
Nanog_Fwd	Sense	TCTTCCTGGTCCCCACAGTTT	qPCR
Nanog_Rev	Antisense	GCAAGAATAGTTCTCGGGATGAA	qPCR
Pou5f1_Fwd	Sense	AGAGGATCACCTTGGGGTACA	qPCR
Pou5f1_Rev	Antisense	CGAAGCGACAGATGGTGG TC	qPCR
Sox2_Fwd	Sense	GCGGAGTGGAAACTTTTGTCC	qPCR
Sox2_Rev	Antisense	CGGGAAGCGTGTACTTATCCTT	qPCR
Pax6_Fwd	Sense	ACATCTTTTACCCAAGAGCA	qPCR
Pax6_Rev	Antisense	GGCAAACACATCTGGATAAT	qPCR
Acrv1b_Fwd	Sense	CTGCCTACAGACCAACTACACC	qPCR
Acrv1b_Rev	Antisense	CCACGCCATCCAGGTTAAAGA	qPCR
Lhx5_Fwd	Sense	AGAACCGAAGGTCCAAAGAA	qPCR
Lhx5_Rev	Antisense	TCACTTTGGTAGTCTCCGTA	qPCR
Ntng2_Fwd	Sense	CAAGGACTCTACGCTTTTCG	qPCR
Ntng2_Rev	Antisense	AGCACTCGCAGTCTTGAAAT	qPCR
Sema3f_Fwd	Sense	CTACACAGCATCCTCCAAGA	qPCR
Sema3f_Rev	Antisense	ACGGCATTCTTGTTTGCATT	qPCR
Smad1_Fwd	Sense	TACTATGAGCTCAACAACCG	qPCR
Smad1_Rev	Antisense	GAAGCGGTTCTTATTGTTGG	qPCR
Smad3_Fwd	Sense	CACGCAGAACGTGAACACC	qPCR
Smad3_Rev	Antisense	GGCAGTAGATAACGTGAGGGA	qPCR
Sox13_Fwd	Sense	CTTACAGGAGGTTGTGCCA	qPCR
Sox13_Rev	Antisense	TCCTTAGCTTCCACATTGCT	qPCR
Stat3_Fwd	Sense	CAATACCATTGACCTGCCGAT	qPCR
Stat3_Rev	Antisense	GAGCGACTCAAACTGCCCT	qPCR
Tcf4_Fwd	Sense	TTGAAGATGTTTTCGCCTCC	qPCR
Tcf4_Rev	Antisense	CCTGCTAGTCATGTGGTCAT	qPCR
Tgfbr2_Fwd	Sense	GAAGGAAAAGAAAAGGGCGG	qPCR
Tgfbr2_Rev	Antisense	TGCTGGTGGTGTATTCTTCC	qPCR
Amylase_Fwd	Sense	GGCTGAGTGTTCTGGGAT	ChIP-qPCR
Amylase_Rev	Antisense	CACGGTGCTCTGGTAGAT	ChIP-qPCR
Cdh1_R1_Fwd	Sense	GCTAGGCTAGGATTCGAACGAC	ChIP-qPCR
Cdh1_R1_Rev	Antisense	TGCAGGGCCCTCAACTT	ChIP-qPCR

**Table 2:** gRNAs and donor template used for CRISPR/Cas9-mediated Zeb2 editing.

Name	Sequence	CRISPR/Cas9
Flag-V5 donor template ^1^	aaaatggaaaccaaatcagaccacgaagaagacaatatggaagatggcatcgaaGACTACAAAGACGATGACGACAAG***gatatc***GGTAAGCCTATCCCTAACCCTCTCCTCGGTCTCGATTCTACG**TAA**actactgcattttaagcttcctattttttttttccagtagtattgtt	in-frame knock-in of Flag-V5 tag
gRNA_ex9_1	GGAAACCAAATCAGACCACGAGG	
gRNA_ΔZP1	CCCGCGCGCGTTTCAATGGGCGC	*Zeb2 ΔP* deletion
gRNA_ΔZP2	CCCTCGCGAGTGCAACACACCAA	
gRNA_ΔZP3	GGGCTCGGAGCGCTGCCGATCGG	
gRNA_ΔZP4	CCGCTGGACCGGGGGGGAGTTGA	

^1^ Flag-V5 donor template (from top left to bottom right): in lowercase: homology arms located in exon9 and 3′ UTR of *Zeb2*, respectively in underlined lowercase: mutated PAM sequences; in uppercase: Flag-encoding sequence; in lowercase italics and bold: *Eco*RI restriction site; in underlined uppercase: V5-encoding sequence; in bold uppercase: STOP codon.

**Table 3:** shRNAs used.

Name	Sequence
shZeb2_1	CCGG**CCGAATGAGAAACAATATCAA**CTCGAGTTGATATTGTTTCTCATTCGGTTTTTG
shZeb2_2	CCGG**CCTCAGGAATTTGTGAAGGAA**CTCGAGTTCCTTCACAAATTCCTGAGGTTTTTG
shZeb2_3	CCGG**CCAGTGTCAGATTTGTAAGAA**CTCGAGTTCTTACAAATCTGACACTGGTTTTTG
shZeb2_4	CCGG**CCCATTTAGTGCCAAGCCTTT**CTCGAGAAAGGCTTGGCACTAAATGGGTTTTTG
shCTRL	CCGGCAACAAGATGAAGAGCACCAACTCGAGTTGGTGCTCTTCATCTTGTTGTTTTT

## Data Availability

The data supporting the reported results will be made available upon request to the corresponding author.

## Supplementary Materials

The following supporting information can be downloaded at https://www.mdpi.com/article/10.3390/genes14030629/s1: Figure S1: Characterization of the Zeb2-V5 mESC lines; Figure S2: Cross-reference of Zeb2-V5-bound protein-coding genes and transcriptome of differentiating mESCs; Figure S3: Top-10 TF motifs enrichment at peak present in −10/+10 kb from the TSS of up or down-regulated genes; Figure S4: Expression of genes resulting from the meta-analysis of Zeb2-bound genes versus three independent RNA-seq datasets; Figure S5: Genotyping of Zeb2ΔP/ΔP mESCs. Figure S6: Distribution of Zeb binding sites (performed as single E-boxes; for details, see main text) and TGFβ/BMP activated (phospho-)Smads and Smad4 binding elements within the identified Zeb2-bound regions for primary target genes; Figure S7. Gene-to-Disease association of the D8 DEGs bound by Zeb2. Table S1: Narrow peaks obtained from the ChIP-seq; Table S2: Transcriptomic data of Zeb2 bound genes in differentiating mESCs; Table S3. Target genes bound by Zeb2 and deregulated in different already published RNA-seq datasets; Table S4. Gene function description of the Zeb2 target genes from Table S3.
