# Supplementary material for: Zeb2 DNA-Binding Sites in Neuroprogenitor Cells Reveal Autoregulation and Affirm Neurodevelopmental Defects, Including in Mowat-Wilson Syndrome
Source: Genes (Basel). 2023 Mar 2;14(3):629. doi: 10.3390/genes14030629 (PMC10048071; doi:10.3390/genes14030629)

Legends to supplementary figures

Figure S1. Characterization of the Zeb2-V5 mESC lines. (**A**) Schematic overview of the design strategy, showing also primers used for genotyping and detection of the *Zeb2* tagged allele; (**B**) Genotyping results (selected part) showing the "heterozygous" band (red arrow) present in mouse (m)ESC clone 2BE3. The lower band represents the wild-type (WT) *Zeb2* allele; (**C**) Zeb2-V5 specific PCR showing the presence of the tagged allele only in clone 2BE3, and not in WT genomic DNA material. Clone 1BD4 was also used as negative control; (**D**) Sanger DNA-sequencing results of clone 2BE3 showing the alignment with the Zeb2V5 sequence designed *in silico*. The last three amino acids (i.e. GME) of WT Zeb2 have been changed to DK to remove a PAM (protospacer adjacent motif) sequence and mutate the remaining PAM sequence, the target of the gRNA, to avoid multiple cutting by Cas9. An *Eco*RI site was inserted between the Flag- and the V5-coding sequences to facilitate the screening, and an artificial STOP codon (indicated by *) was added downstream of the V5-coding sequence.

Figure S2. Cross-reference of Zeb2-V5-bound protein-coding genes and transcriptome of differentiating mouse (m)ESCs. (**A**) Volcano plots showing the distribution of Zeb2-bound genes in the whole transcriptomes of day (D) D4, D6 and D8 neural differentiated mESCs. Red dots depict the genes bound by Zeb2 and grey crosses those not bound; (**B**) About 11 to 14% of the up or down-regulated genes are bound by Zeb2 (red part of the bars) at D4-D8; (**C**) Of the 1,952 genes bound by Zeb2 and found being differentially expressed genes (DEGs) during mESC differentiation, 335 are in common among the three considered time points. *Zeb2* itself is among these common genes and its mRNA expression increases during differentiation (yellow dot). The volcano plots show the distribution of the common genes during differentiation; (**D**) Time point specific DEGs depicted as volcano plots. The grey arrow in D8 panel indicates *Tcf4*.

Figure S3. Top-10 transcription factor (TF) motifs enrichment at peak present in -10/+10 kb from the TSS of up or down-regulated genes. Scatter plots represent the top-10 TF motifs found in the peaks present in the -10/+10 kb from the TSS range in genes which are up or down-regulated during mouse ESC neural differentiation (panel **A** and **B**, respectively). Each dot represents a peak. Enrichment analysis was performed using UniBind (see Materials and Methods section).

Figure S4. Expression of genes resulting from the meta-analysis of Zeb2-bound genes versus three independent RNA-seq datasets. The heatmap visualizes the log2FoldChange of the resulting genes during mouse (m)ESC differentiation. RNA-seq datasets where the genes have been found to be differentially expressed are annotated as green (Nkx2.1-Cre|Zeb2 mouse model), purple (Zeb2 knockdown (KD) in mESCs) or yellow (Gsh2-Cre|Zeb2 mouse model) squares.

Figure S5. Genotyping of $Zeb2^{\Delta P/\Delta P}$ mouse (m)ESCs. (**A**) Schematic overview of the binding sites for the primers (listed in Table 1) used for genotyping; (**B**) PCR confirming the proper deletion of the selected region in ESC clone E9; (**C**) Further PCR showing the difference in fragment length for regions amplified with different sets of primer and discriminating between WT, heterozygous ($Zeb2^{\Delta p/+}$) and homozygous ($Zeb2^{\Delta p/\Delta p}$) deletion ESC clones; (**D**) Sanger sequencing showing the correct deletion of the selected region in the $Zeb2^{\Delta p/\Delta p}$ clone.

Figure S6. Distribution of Zeb binding sites (performed as single E-boxes; for details, see main text) **and TGFβ/BMP-activated (phospho-)Smad and Smad4 binding elements within the identified Zeb2-bound regions for primary target genes.** (**A**) Grey triangles represent distance from the transcription start site (TSS), while blue triangles depict the TSS of the individual genes. Direction of the arrow shows also whether the gene is transcribed on the + or – strand; (**B**) Motif analysis identifies the binding motif for Zeb2 as CACCTG. Analysis of the distribution and motif analysis were performed using the Jasper database with a confidence score >80% (https://jaspar.genereg.net).

Figure S7. Gene-to-Disease association of the D8 DEGs bound by Zeb2. The gene names of the differentially expressed genes (DEGs) expressed at D8 of ESC neural differentiation were initially subjected to a mouse-to-human gene conversion using biomaRt package for R. After that, gene-to-disease association analysis was performed using disgenet2r package and API dependency, with an association score >30% to retrieve Gene to Disease association from DisGeNET (https://www.disgenet.org/disgenet2r).

Supplementary figure 1

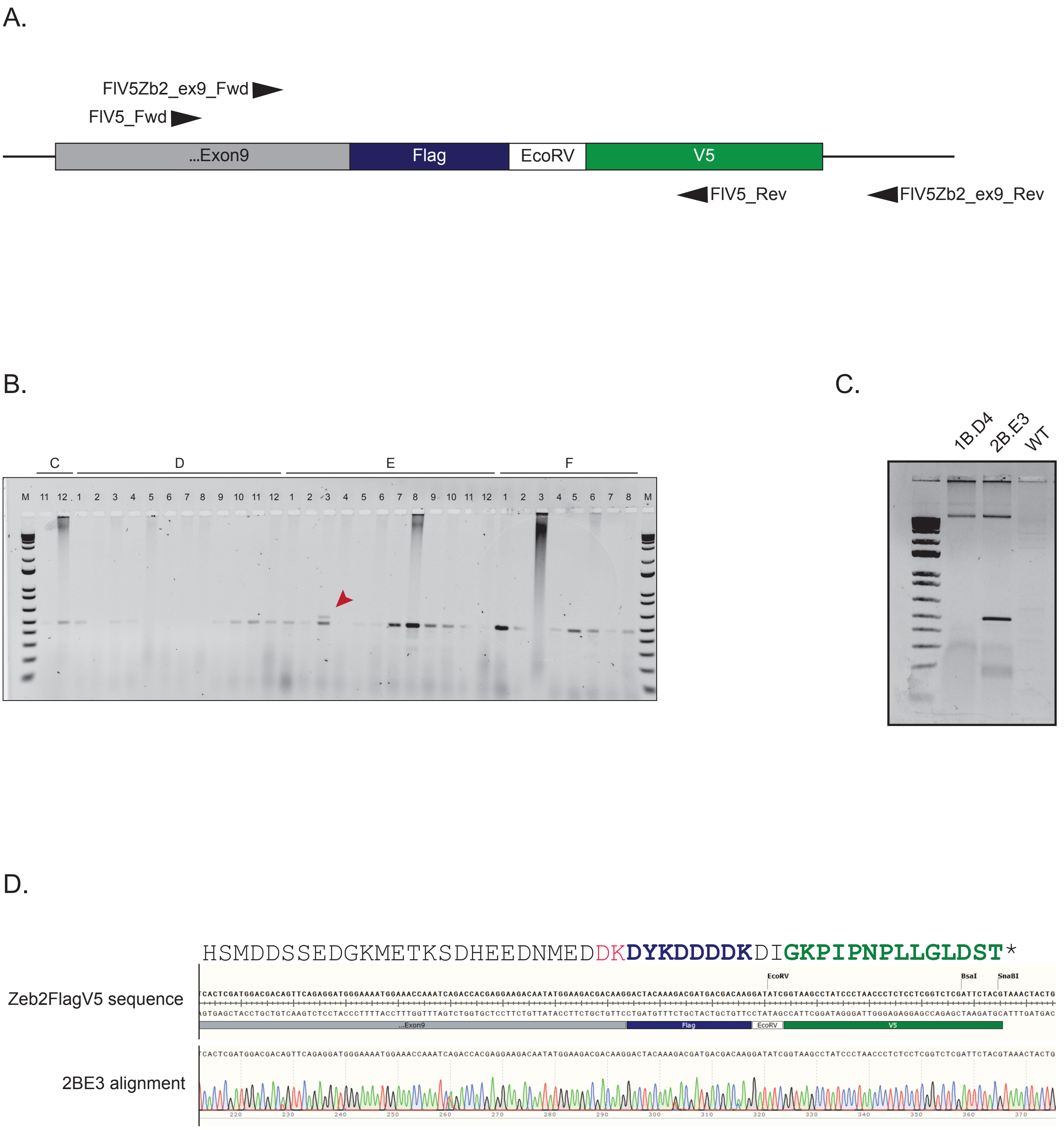

Supplementary Figure 2

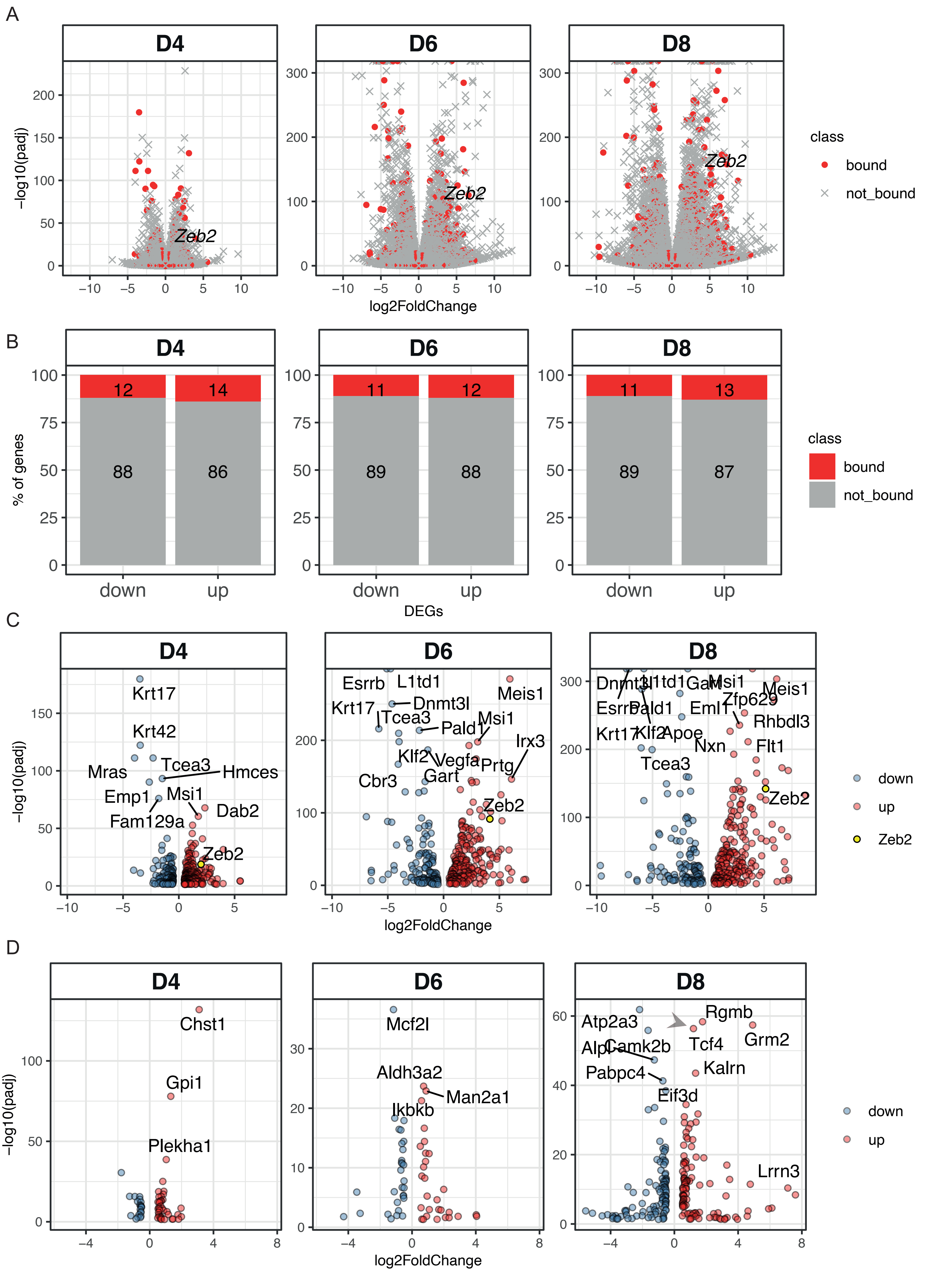

A

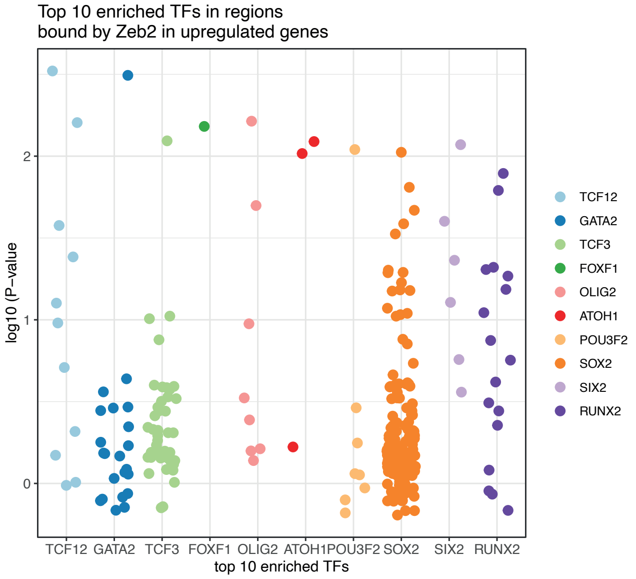

B

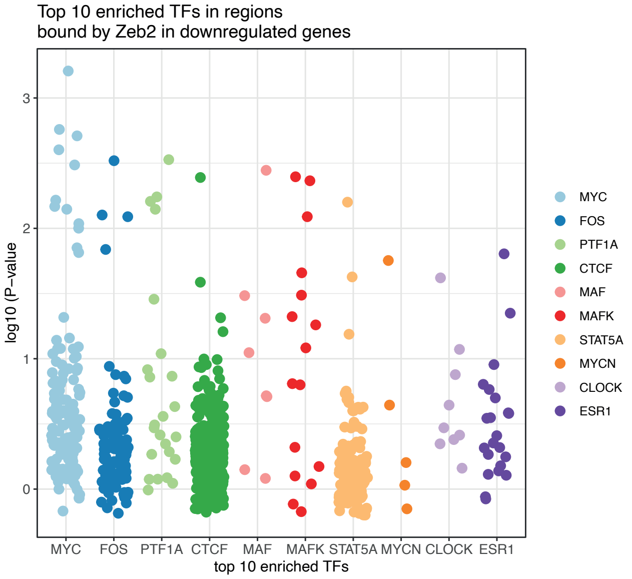

Birkhoff et al 2021_
Zeb2V5 ChIPseq

Supplementary Figure 4

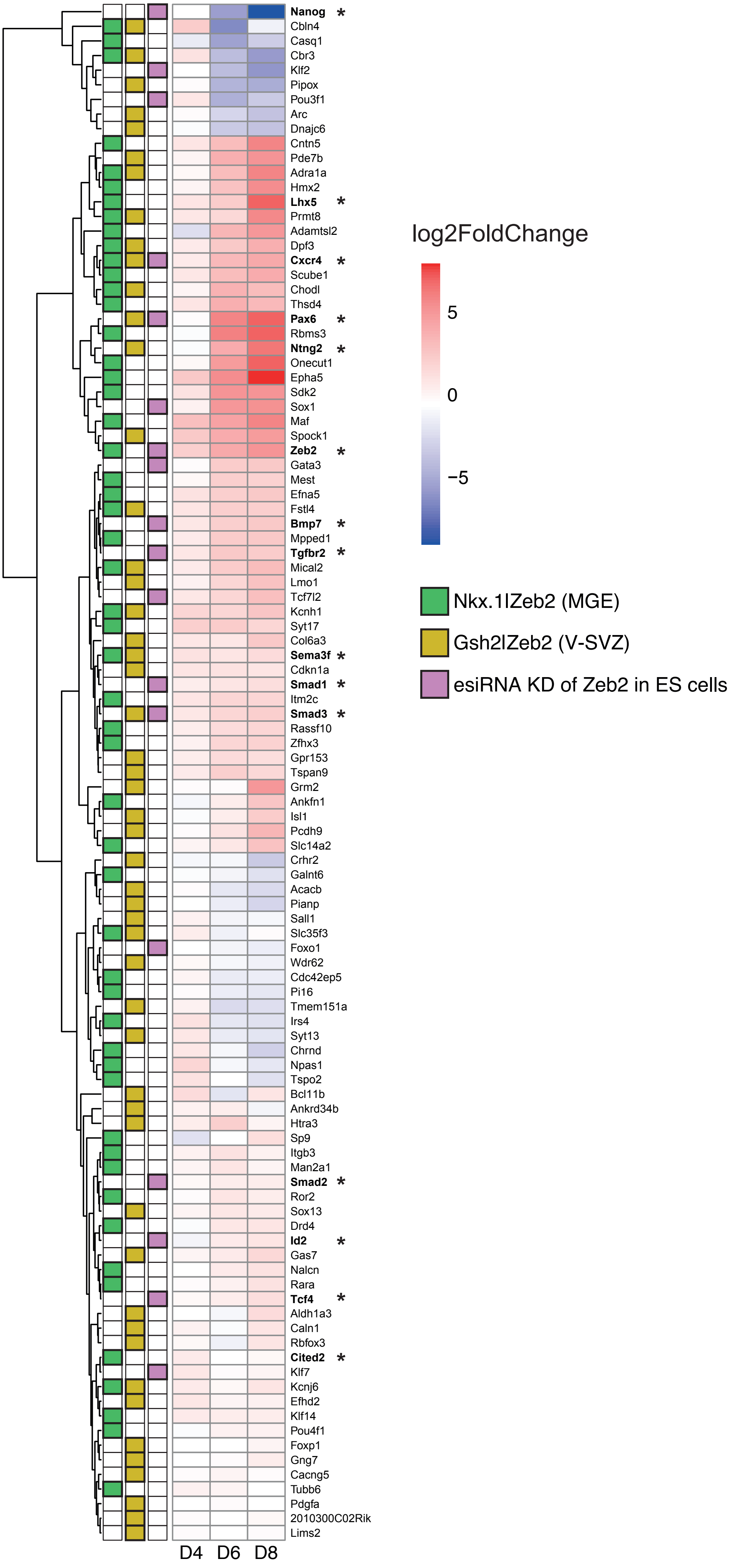

A.

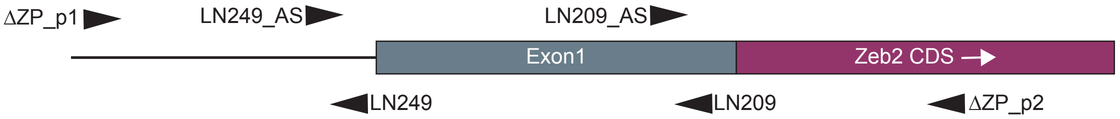
ΔZP_p1
LN249_AS
LN209_AS
Exon1
Zeb2 CDS
LN249
LN209
ΔZP_p2

B.

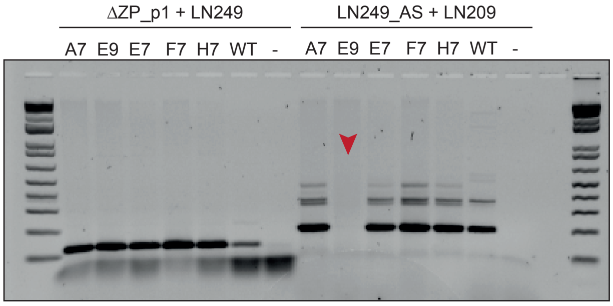
ΔZP_p1 + LN249
LN249_AS + LN209
A7 E9 E7 F7 H7 WT -
A7 E9 E7 F7 H7 WT -

C.

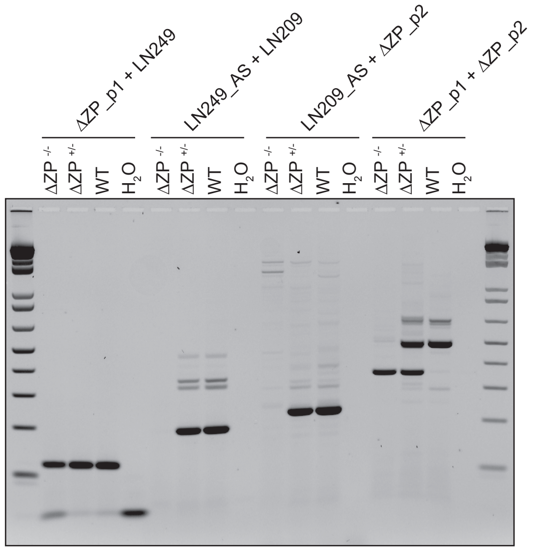
ΔZP_p1 + LN249
LN249_AS + LN209
LN209_AS + ΔZP_p2
ΔZP_p1 + ΔZP_p2
ΔZP -/-
ΔZP +/-
WT
H2O

D.

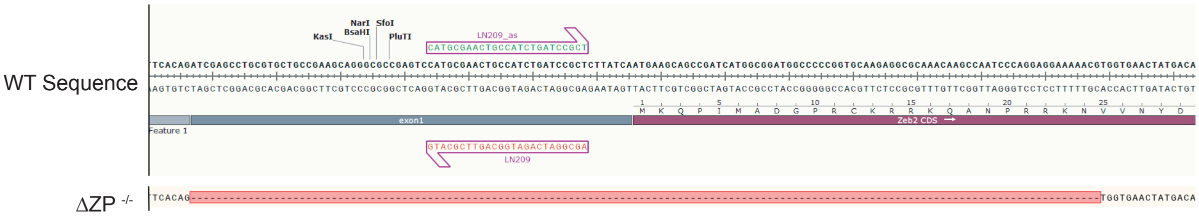
WT Sequence
ΔZP -/-
LN209_as
exon1
Zeb2 CDS
Feature 1
LN209

A

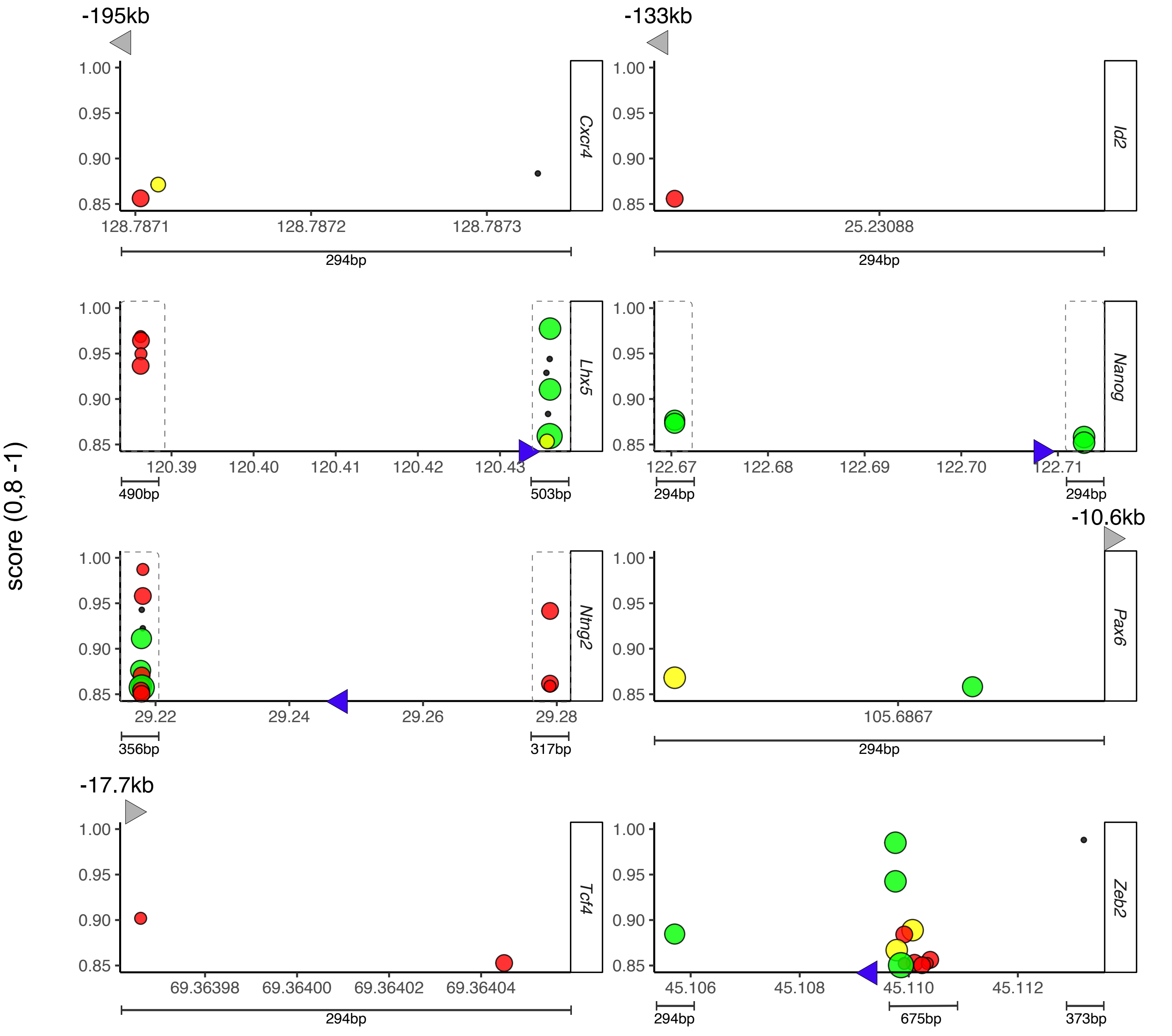

-195kb
-133kb
-10.6kb
-17.7kb
score (0,8 -1)
1.00
0.95
0.90
0.85
Cxcr4
Id2
Lhx5
Nanog
Ntng2
Pax6
Tcf4
Zeb2
128.7871
128.7872
128.7873
25.23088
120.39
120.40
120.41
120.42
120.43
122.67
122.68
122.69
122.70
122.71
29.22
29.24
29.26
29.28
105.6867
69.36398
69.36400
69.36402
69.36404
45.106
45.108
45.110
45.112
294bp
490bp
503bp
356bp
317bp
675bp
373bp
Zeb1/Zeb2
TGFβ R-Smads
BMP R-Smads
Smad4

B

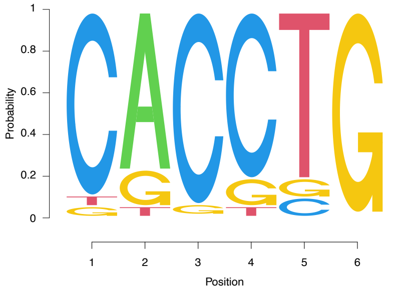

Probability
Position

Supplementary Figure 7

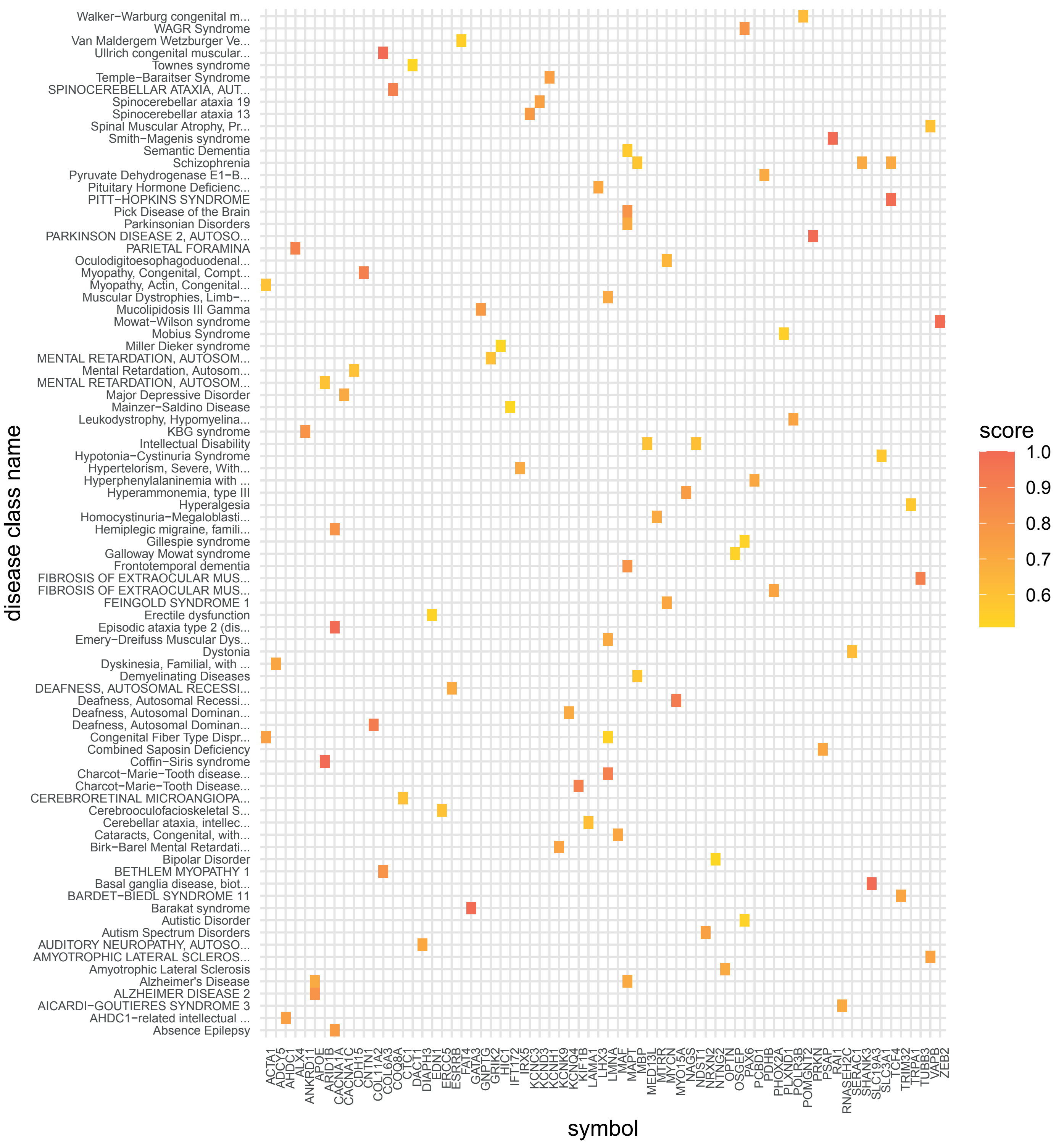

Supplement: Supplementary file 1 [file genes-14-00629-s001.zip › Supplementary Figures_R1.pdf]
